# A randomised controlled trial of amygdala fMRI-neurofeedback versus sham-feedback in borderline-personality disorder – systematic literature review and introduction to the BrainSTEADy trial

**DOI:** 10.1186/s12888-025-07000-1

**Published:** 2025-07-08

**Authors:** Christian Paret, Miroslava Jindrová, Nikolaus Kleindienst, Judith Eck, Hester Breman, Michael Lührs, Beatrix Barth, Thomas Ethofer, Andreas J. Fallgatter, Rainer Goebel, Andreas Hoell, Denise Lockhofen, Annika S. Reinhold, Simon Maier, Swantje Matthies, Christoph Mulert, Christian Schönholz, Ludger Tebartz van Elst, Christian Schmahl

**Affiliations:** 1 Department of Psychosomatic Medicine and Psychotherapy, Central Institute of Mental Health, Medical Faculty Mannheim/Heidelberg University, Mannheim, Germany; 2 German Center for Mental Health, Partner Site Mannheim-Heidelberg-Ulm, Heidelberg, Germany; 3 Brain Innovation B.V., Research Department, Maastricht, The Netherlands; 4 Department of Cognitive Neuroscience, Faculty of Psychology and Neuroscience, Maastricht University, Maastricht, The Netherlands; 5 Department of Psychiatry and Psychotherapy, Tuebingen Center for Mental Health, University Hospital Tuebingen, Tuebingen, Germany; 6 German Center for Mental Health (DZPG), Partner Site Tuebingen, Heidelberg, Germany; 7 Department of Psychiatry and Psychotherapy, Central Institute of Mental Health, Medical Faculty Mannheim/University of Heidelberg, Mannheim, Germany; 8 Centre of Psychiatry, Justus-Liebig University, Klinikstrasse 36, 35392 Giessen, Hessen Germany; 9 Department of Public Mental Health, Central Institute of Mental Health, Medical Faculty Mannheim/Heidelberg University, Mannheim, Germany; 10 Department of Psychiatry and Psychotherapy, Medical Center - University of Freiburg, Faculty of Medicine, University of Freiburg, Freiburg, Germany; 11 Centre for Mind, Brain and Behaviour (CMBB), Hans-Meerwein-Strasse 6, 35043 Marburg, Hessen Germany

**Keywords:** Amygdala, Neurofeedback, Borderline-personality disorder, Randomized-controlled trial, Emotion regulation, Ecological momentary assessment, Brain-computer interface, Neuroimaging, Systematic literature review, Safety

## Abstract

**Background:**

Individuals with Borderline-Personality Disorder (BPD) experience intensive, unstable negative emotions. Hyperactivity of the amygdala is assumed to drive exaggerated emotional responses in BPD. Functional Magnetic Resonance Imaging (fMRI)-based neurofeedback is an endogenous neuromodulation method intended to address the imbalance of neural circuits and thus holds the potential as a treatment for BPD. Many original articles and meta-analyses show that fMRI-neurofeedback can improve psychiatric symptoms. In contrast, there is a lack of publications that aggregate and evaluate data of the safety of the treatment. Furthermore, evidence on the efficacy of fMRI-neurofeedback for the treatment of BPD is limited. Preliminary evidence suggests that downregulation of amygdala hyperactivation through fMRI-neurofeedback can ameliorate emotion dysregulation. To test this assumption, BrainSTEADy (Brain Signal Training to Enhance Affect Down-regulation), a multi-center clinical trial, is conducted. First, we present a systematic literature review evaluating the safety of fMRI-neurofeedback and assessing clinical performance in BPD. Second, we describe the study protocol of BrainSTEADy.

**Methods:**

Literature research: From 2,609 screened paper abstracts, 758 were identified as potentially relevant. Twenty studies reported adverse events or undesirable side effects. Two papers provided relevant data for the assessment of clinical performance in BPD. BrainSTEADy study protocol: During four sessions, patients will receive graphical fMRI-neurofeedback from their right amygdala or sham-feedback while viewing images with aversive content. The primary endpoint, ‘negative affect intensity’, will be assessed after the last neurofeedback session using Ecological Momentary Assessment (EMA). Secondary endpoints will be assessed after the last neurofeedback session, at 3-month and at 6-month follow-up. This trial is a multi-center, patient- and investigator-blind, randomized, parallel-group superiority study with a planned interim-analysis once half of the recruitment target is met (*N* = 82).

**Discussion:**

As suggested by literature review, fMRI-neurofeedback is a safe treatment for patients, although future studies should systematically assess and report adverse events. Although fMRI-neurofeedback showed promising effects in BPD, current evidence is limited and calls for a randomized controlled trial such as BrainSTEADy, which aims to test whether amygdala-fMRI-neurofeedback specifically reduces emotion instability in BPD beyond nonspecific benefit. Endpoint measures encompassing EMA, clinical interviews, psychological questionnaires, quality of life, and neuroimaging will enable a comprehensive analysis of effects and mechanisms of neurofeedback treatment.

**Trial registration:**

The study protocol was first posted 2024/10/04 on ClinicalTrials.gov and received the ID NCT06626789.

**Supplementary Information:**

The online version contains supplementary material available at 10.1186/s12888-025-07000-1.

## Background

Individuals with Borderline-Personality Disorder (BPD) experience highly negative emotions [8] and are characterized by emotional instability [8]. At the brain level, hyperactivity of the amygdala in response to pictures with emotional content has been observed [39]. Current psychobiological models of BPD postulate an imbalance between hypersensitive emotional brain systems such as the amygdala and hypoactive ‘emotion-regulation systems’, comprising the medial and dorsolateral prefrontal cortex [39]. This imbalance makes individuals with BPD vulnerable to intense emotions, for which they attempt to compensate with dysfunctional regulation behaviors (e.g. non-suicidal self-injury), leading to frequent medical treatment, social turbulences and negative emotions against the self [3].

Psychotherapy is considered the first-line treatment for BPD [3, 44]. BPD-specific psychotherapy programs are more effective as compared to standard psychotherapy treatment for the improvement of symptom severity, psychosocial functioning, self-injurious and suicidal behaviours [44]. Still, a significant number of patients either drops out prematurely or does not sufficiently respond to BPD-specific psychotherapy [45]. Therefore, there is a great demand to improve existing treatments and to develop new treatment options for these patients. One promising candidate for novel treatments is neurofeedback, which could e.g. be an add-on treatment to psychotherapy in the future. Neurofeedback enables persons to self-regulate their brain activation using real-time feedback from the brain. Electroencephalography (EEG)-based neurofeedback is an established medical procedure and has been used for decades for treating various mental disorders [46]. However, EEG, in contrast to functional Magnetic Resonance Imaging (fMRI), lacks the anatomical precision to measure activation of deep-brain regions such as the amygdala. As individuals with BPD show an exaggerated amygdala response to negative images [39], fMRI-neurofeedback seems more promising than EEG-Neurofeedback. Real-time fMRI-based neurofeedback has only become available for neurofeedback training around the beginning of the millennium [46]. An increasing number of studies are investigating the effectiveness of fMRI-neurofeedback to train emotion regulation, both in healthy samples and in patient populations [25]. Multiple studies have specifically targeted the amygdala to treat disorders of emotion and mood, such as BPD [51], Posttraumatic Stress Disorder [32, 53] and Major Depressive Disorder (MDD) [50]. A meta-analysis across nine studies comparing amygdala-neurofeedback with control treatment found a high aggregated effect size (Cohen’s d = 0.75) confirming improved amygdala regulation in treatment versus control groups [11]. Additionally, meta-analyses suggest the potential superiority of amygdala-targeted neurofeedback versus placebo across various psychiatric indications [11]. Corroboration of these findings by more randomized controlled trials is necessary. In a four-session fMRI amygdala-neurofeedback training, administered to eight female patients with BPD, we observed down-regulation of amygdala activation and increased functional connectivity between the amygdala and prefrontal cortex [34]. This initial study demonstrated feasibility and showed that patients tolerated neurofeedback well. A second study extended previous findings [51]. In this study we investigated which aspects of emotion dysregulation would be malleable by neurofeedback. Twenty-five female BPD patients participated in three neurofeedback sessions and were tested again six weeks later. For inclusion, patients needed to be on constant medication or outpatient treatment throughout the study period. Emotion regulation was assessed on the physiological, behavioral and self-report level. After training, patients reported reduced negative affect intensity and an improvement in symptoms. In the psychophysiology lab, patients revealed improved emotion regulation skills after training, indicated by decreased startle responses to negative pictures. This second study revealed significant improvement in emotion regulation and reductions in affect intensity in daily life. Conclusions are limited due to the lack of control groups in these studies. An ongoing open-label trial (clinicaltrials.gov: NCT04333888) aims to assess the feasibility and neurofeedback effects in a psychotherapy setting including a treatment-as-usual control group. While this research will inform potential clinical effects, it is not designed to show specificity of amygdala-neurofeedback (vs. a comparison treatment).

Taken together, current evidence supports amygdala self-regulation using neurofeedback as a potential mechanism to ameliorate BPD symptoms, in particular overwhelming negative affect. Pilot studies in BPD indicate that neurofeedback has the potential to change emotion processing at several levels, including psychophysiology, behavior and subjective experience. However, randomized controlled trials investigating the effectiveness of real-time fMRI neurofeedback treatment in BPD are lacking.

In the first part of this paper, we report results from a systematic literature review to evaluate safety, clinical performance and clinical benefits of fMRI-neurofeedback. The second part describes a randomized clinical trial designed to overcome the lack of evidence and to determine whether amygdala fMRI-neurofeedback has a specific effect on the intensity of negative affect in BPD beyond nonspecific benefit.

## Systematic literature review

### Methods

The literature search aimed to aggregate information from existing studies to evaluate the safety, clinical performance, clinical benefit and the state of the art of fMRI-neurofeedback. We report the general results regarding safety and clinical performance, and about clinical benefits for patients diagnosed with BPD.

#### Database search strategy

A systematic literature search was conducted on October 10, 2023, using PubMed (MEDLINE), Web Of Science (Core Collection), Embase, Cochrane, and ClinicalTrails.gov. The search was updated on April 30, 2024 (Table S2). The resulting items were imported to the browser app Rayyan [33] for further processing.

#### Abstract review

Following automatic and manual duplicate removal, all abstracts were reviewed in light of the research questions: (1) Is real-time fMRI-neurofeedback safe? (2) What is the current evidence on clinical performance of fMRI-neurofeedback for BPD? (3) What is the state of the current literature regarding clinical benefits of fMRI-neurofeedback for BPD? After cross-checking and screening the search results, 707 publications from the initial search and 51 publications from the updated search were identified as potentially relevant. The abstract review procedure is described below in further detail.

In a first non-blinded review round each record was assessed by a single reviewer (HB, JE) for relevance. Records matching the following criteria were excluded:

1. Undetected duplicates from the duplicate removal stage or duplicates with records of the previous literature search round
2. Publications with non-human study population
3. General background articles not focusing specifically on fMRI-neurofeedback
4. Publications written in a language other than English or German
5. General methods paper not focusing on fMRI-neurofeedback
6. Publications not focusing on fMRI-neurofeedback
7. Wrong publication types

If more than one reason was chosen for a specific record, only the major reason was kept as exclusion label for the final decision. In a second abstract approval round, all records marked as potentially relevant were labeled by the reviewer who had not evaluated the record in the first round. Additionally, all records that were labeled as potentially relevant for the state-of-the-art were evaluated by a third expert reviewer (ML). Disagreements regarding relevance were resolved by consensus and 154 abstracts were excluded at this stage.

Three additional searches on clinicaltrials.gov were performed on December 7, 2023 to obtain trials that were not labeled as randomized controlled trial ([RCT]; because the previous search was limited to RCTs), using the following search terms:

- (Borderline(Condition)) AND (fmri AND neurofeedback (Other terms))

On the same day, also searches were performed for other conditions:

- ((Depression(Condition)) AND (fmri AND neurofeedback (Other terms)); (Parkinson's Disease OR Parkinson OR Parkinson Disease(Condition)) AND (fmri AND neurofeedback (Other terms))).

All clinical trials from this search that were not already included were added to the abstract review round. Among the 17 trials that were added at this stage, four trials addressed BPD. In total, 2,399 records were screened during the initial review stage, and 210 records during the update. Of these, 1,692 records were excluded in the first round and 707 records were labeled as potentially relevant. In the update round, 159 records were excluded and 51 were labeled as potentially relevant. The complete list of reviewed publications based on the abstract information can be found in Table S3. Each of the potentially relevant publications was labeled according to the publication type (Table S4) and clinical population (Table S5). This information was used to initiate two separate full text reviews, as described below. Thirteen of the potentially relevant publications focused on BPD.

#### Fulltext screening: adverse events and undesirable effects

Original research publications (*N* = 365) and meta-analyses (*N* = 16) were screened for reported adverse events, undesirable side-effects and dropouts that were potentially related to the general fMRI-neurofeedback procedure (Table S6).

#### Fulltext screening: selection and evaluation

Full text articles were evaluated regarding their relevance and clinical significance to the research questions. Publications were considered irrelevant if:

- No results were reported
- The data, more specifically the results, were already reported in another publication
- Publications were not using fMRI-neurofeedback for treating BPD

The criteria used to evaluate the quality and relevance are described in Table S7.

#### Fulltext screening: analysis

**Fig. 1:**
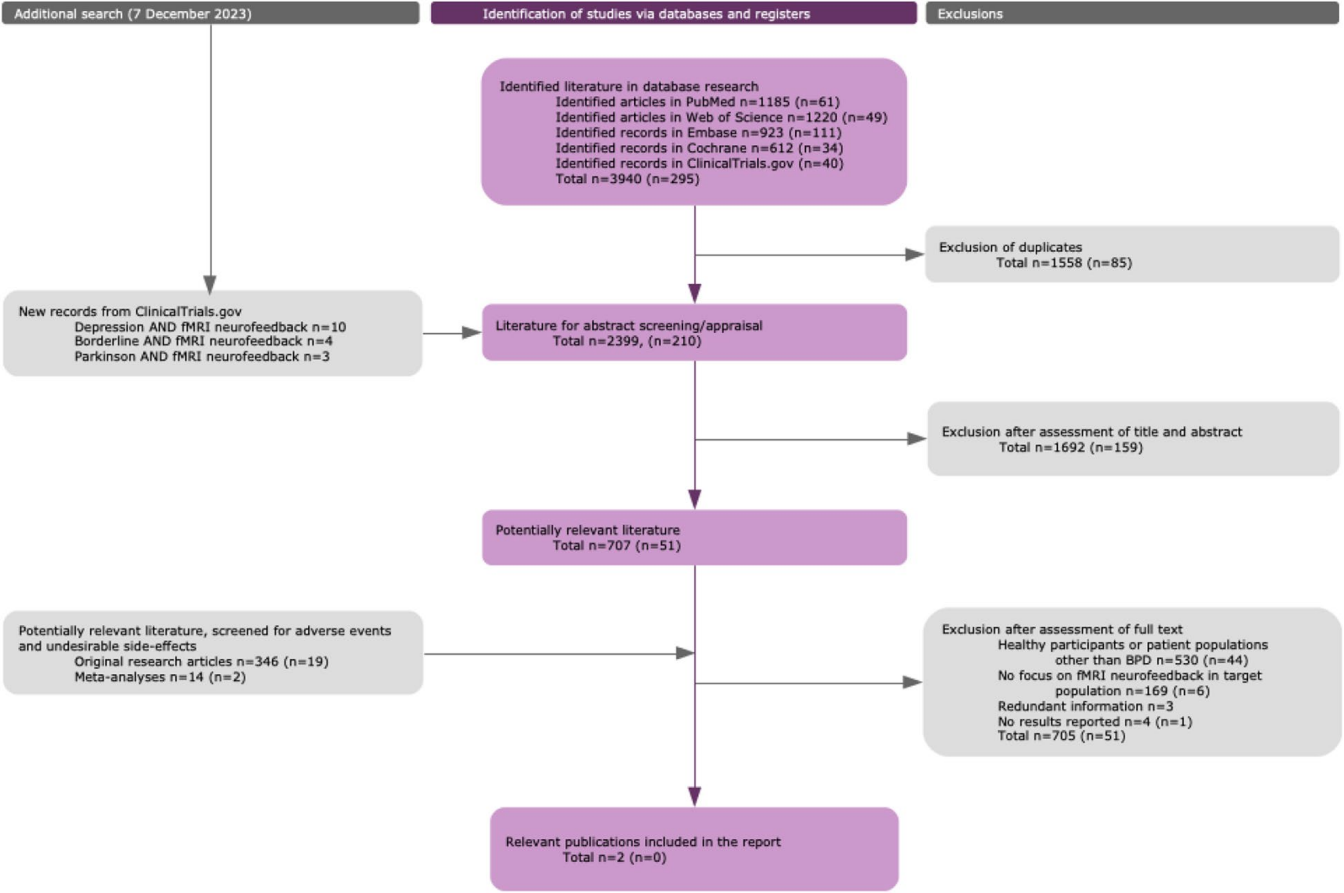
Literature search protocol. Note: The literature search was part of a large project that, first, addressed safety and state-of-the-art of fMRI-neurofeedback in general, i.e., across populations, and second, focussed on three different diagnostic entities, i.e., BPD, MDD and Parkinson’s disease. In this article, we report the general results and the BPD-specific results. For consistency, the non-relevant diagnostic entities are still included in the methods

Thirteen of the potentially relevant publications focused on patients with BPD. Eleven of these publications were subsequently excluded due to lack of focus on fMRI-neurofeedback, redundant information or missing results. The remaining two publications were analyzed with respect to performance, safety, clinical benefit and undesirable side-effects. The full evaluation of the two relevant publications can be found in Table S8. The flowchart of the literature search is shown in Fig. 1.

### Results

Neither of the two publications of fMRI-neurofeedback in BPD reported undesirable side-effects or any adverse events. However, it should be noted that such information was not systematically assessed in these studies. Twenty studies of all 381 potentially relevant original research publications and meta-analyses in various mental conditions reported undesirable side effects or adverse events, the majority of which were mild and often occurred in the control-group not receiving neurofeedback (Table S9). There was one nonlethal overdose of paracetamol in a study with alcohol dependent participants, which occurred in the control group not receiving neurofeedback (i.e., treatment as usual).

Both original publications reporting results from fMRI-neurofeedback in BPD used a trial design without a control group. Paret et al. [34] intended to assess the feasibility of fMRI-neurofeedback with patients, while Zaehringer et al. [51] assessed effect sizes of change in emotion-regulation-related outcomes achieved through real-time fMRI-neurofeedback. Both publications showed that BPD patients were able to down-regulate the Blood Oxygenation Level Dependent (BOLD)-response in the target area, while improvement of regulation over time was not statistically significant (improvement over time would suggest that patients learned). Paret et al. [34] showed improvements in dissociation and ‘lack of emotional awareness' over the course of four neurofeedback sessions. However, these findings should be interpreted with caution due to the small sample size. Zaehringer et al. [51] showed clinical improvements in emotion dysregulation, BPD psychopathology and affective instability, but stressed that the results are 'preliminary and should be replicated by an independent study’, since the results did not remain significant after correction for multiple comparisons. At the time of the literature search, three registered clinical trials on amygdala-regulation in BPD were ongoing, two employed downregulation of the BOLD response (NCT04306341 and NCT04333888) and one assessesed the effect of amygdala-upregulation in response to positive memories (NCT05398627).

## BrainSTEADy trial protocol

### Aim of trial and general description of design

BrainSTEADy (Brain Signal Training to Enhance Affect Downregulation) is a randomized controlled, patient-, investigator-, and biometrician-blinded, multi-center trial to assess the superiority of fMRI-neurofeedback compared to sham-control feedback in treating BPD. The study is designed to confirm the effects of fMRI-neurofeedback treatment on negative affect intensity (i.e., primary endpoint) and emotion dysregulation (Table 1, for trial synopsis see Table S1 in the Supplement). Further aims of the investigation are to assess neurocognitive mechanisms, the cost-effectiveness, and the influence of pathological states (including dissociation) that may influence treatment response.

**Table 1:** Trial registration dataset

Data category	Information
Primary registry and trial identifying number	ClinicalTrials.gov, NCT06626789
Date of registration in primary registry	2024, October 4
Secondary identifying numbers	none
Source(s) of monetary or material support	German Research Foundation (DFG)
Primary sponsor	Central Institute of Mental Health, Mannheim, Germany
Secondary sponsor	none
Contact for public queries	christian.paret@zi-mannheim.de
Contact for scientific queries	christian.paret@zi-mannheim.de
Public title	Brain Signal Training to Enhance Affect Down-regulation—BrainSTEADy
Scientific title	A multi-center, patient-blind and investigator-blind, randomized, parallel-group, superiority study to investigate a neurobiological mechanism of affect instability, comparing four sessions of amygdala fMRI-BOLD neurofeedback with sham-feedback in Borderline Personality Disorder
Countries of recruitment	Germany
Health condition(s) or problem(s) studied	Borderline-Personality Disorder (BPD)
Intervention(s)	Active comparator: fMRI-Neurofeedback
	Placebo comparator: Sham-feedback
Key inclusion and exclusion criteria	Ages eligible for study: 18–65
	Inclusion criteria: BPD Diagnosis, insufficient response to ≥ 2 therapies
	Exclusion criteria: Alcohol/substance dependence, psychotic disorder, MRI-exclusion criteria
Study type	Interventional
	Allocation: randomized; Intervention model: parallel assignment; Masking: investigator-, patient-, biometrician blinded
	Primary purpose: symptom improvement
	Phase II
Date of first enrolment	Apr 25
Target sample size	164 (82)
Recruitment status	Recruiting
Primary outcome	Negative affect intensity (time frame: immediately after intervention)
Key secondary outcomes	Negative affect intensity (time frame: 3 months after intervention), Borderline symptom severity (time frames: immediately after intervention, 3 months after intervention)

**Fig. 2:**
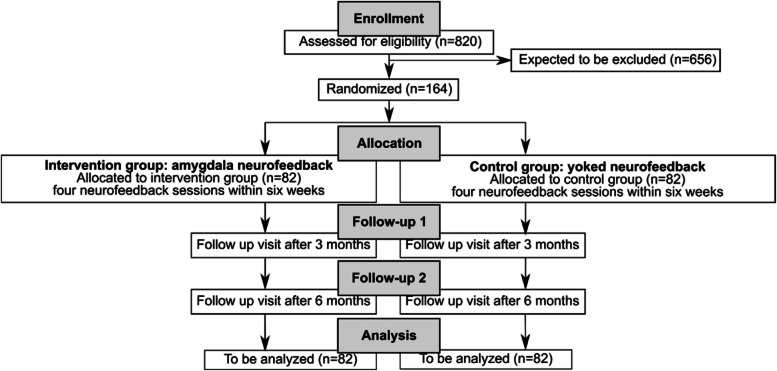
Visualization of trial design with patient time flow

Patients visit the study centers for four sessions of either amygdala-feedback or a control treatment. The primary endpoint, ‘negative affect intensity’, will be assessed with EMA before and after treatment. Secondary endpoints will be assessed immediately following treatment and during two additional time points to follow up improvements (3-months, 6-months after primary endpoint assessment) (Table 2). The trial will be conducted at four university hospitals in Germany. Participants will be randomized 1:1 to receive either the experimental or the control condition. Participants receiving the control treatment will not get feedback from their own brain activation. Instead, the signal of another participant will be presented, a procedure also known as ‘yoked feedback’. Blinding of group assignment will be implemented by the neurofeedback software based on a randomization code entered by the investigator at the beginning of the neurofeedback session, with the randomization list hidden from patients and investigators. Group assignment will remain concealed for the patient, the investigator and the biometrician until the event of debriefing and data analysis, respectively. An interim analysis will be carried out after half of the subjects have been assessed. If the clinical investigation continues without adaptation, the final analysis will be performed after the inclusion of 164 subjects (Fig. 2). Recruitment started in April 2025 (first subject in), the recruitment phase is planned for 2 years (last subject out in March 2027). At the time of publication of this manuscript, the final study protocol, i.e., the Clinical Investigation Plan (CIP), approved by the ethics committee was V02, dated 2025, March 10. Subjects will be recruited at four German university clinics, i.e., University Hospital Freiburg, Justus-Liebig University Giessen, Central Institute of Mental Health in Mannheim, and University Hospital Tuebingen. Patients will be recruited from the local outpatient and inpatient clinics, via social media, the study webpage www.neurofeedback-borderline.de, and leaflets distributed to external psychiatric and psychotherapeutic services. Patients will be reimbursed per completed baseline, post-assessment, and follow-up visit.

**Table 2:** Schedule of assessments

Schedule of assessments										
Week	0	0–4			2–5	3–6	4–7	5–10	18–22	30–34
Visit	V1	V2	V3	V4				V5	FUP1	FUP2
Description	Screening, Baseline part 1	Baseline part 2, Neurofeed-back 1	Neurofeed-back 2	Neurofeed-back 3	Interim week 1	Interim week 2	Interim week 3	Neurofeed-back 4, Post-assessment (T1)	Follow-up (T2)	Follow-up (T3)
**Procedures ↓**										
Informed consent, eligibility assessment:										
• Structured Clinical Interview for DSM-5 (SCID) • International Personality Disorder Examination (IPDE) • Columbia-Suicide Severity Rating Scale (C-SSRS) • Medical history, concomitant medication, demography	x									
Randomisation		x								
Pregnancy test, if applicable		x	x	x				x		
Menstruation assessment, if applicable	x	x	x	x				x		
Magnetic Resonance Imaging:										
• Neurofeedback		x	x	x				x		
• Diffusion Tensor Imaging		x	x					x		
Ecological Momentary Assessment (EMA)										
• Negative affect • Positive affect • Activity • Negative experiences • Positive experiences • Aversive tension • Emotion control • Interpersonal events		x						x	x	
AE/SAE		x	x	x				x	x	
**Questionnaires & interviews ↓**										
Assessment of Quality of Live (AQoL-6D)		x								x
Borderline Symptom List (BSL-23)	x	x						x	x	x
Beck Depression Inventory (BDI-II)	x							x	x	x
Blind check^a^								x		
Clinical Global Impression (CGI)	x							x		
Concluding questionnaire^a^								x		
Difficulties in Emotion Regulation Scale (DERS-36)	x							x	x	x
Dissociative Experiences Scale (DES)	x									
Dissociation-Tension Scale 4-item version (DSS-4)		x	x	x				x		
Dissociation-Tension Scale acute (DSS-acute)		x						x	x	x
Fragebogen zur Inanspruchnahme medizinischer und nicht medizinischer Versorgungsleistungen bei psychischen Erkrankungen (FIMPsy)	x									x
Mental Strategy Questionnaire for Neurofeedback (MSQ-NF)		x	x	x				x		
Paranoia Checklist (PCL)		x								
Relationship Scales Questionnaire (RSQ)		x								
Symptoms Checklist 27 (SCL-27)	x							x	x	x
Survey of Neurofeedback transfer^a^					x	x	x	x		
Work Productivity and Activity Impairment Questionnaire: General Health (WPAI:GH)	x									x
Zanarini Rating Scale for Borderline-Personality Disorder (ZAN-BPD)	x							x	x	

^a^See Online Supplement

### Patient eligibility

#### Inclusion criteria

Subjects meeting all of the following criteria will be considered for enrollment in the clinical investigation:

1. Age 18–65 years
2. Diagnosis of BPD according to DSM-5
3. Insufficient response to ≥ 2 therapies. The criterion is fulfilled if the patient reports:
  - 2 or more psychotherapy treatments with at least 12 sessions each, OR:
  - 2 or more psychotherapy treatments, each with a duration of at least 12 weeks OR:
  - a medical history of 2 or more psychopharmacological treatments, each over the course of at least 4 weeks OR:
  - a combination of 2 or more treatments such as:
    psychotherapy with at least 12 sessions,
    psychotherapy with a duration of at least 12 weeks,
    psychopharmacological treatment of at least 4 weeks duration.
4. Sufficient German language skills to give informed consent to the study, to understand questions posed by the instruments used
5. Ability to understand the nature and individual consequences of clinical investigation
6. Written informed consent (mandatory before enrollment in the clinical investigation)
7. Adequate contraception for women of childbearing potential.

#### Exclusion criteria

Subjects presenting with any of the following criteria will not be included in the clinical investigation:

1. Treatment with benzodiazepines within 7 days prior the initial screening
2. Current alcohol or substance dependence (i.e., within 1 month prior the initial screening)
3. Meeting the diagnostic criteria for a psychotic disorder or schizophrenia (life-time), as determined by clinical interview at initial screening
4. Current or history of significant neurological condition (such as stroke, traumatic brain injury, space occupying lesions, multiple sclerosis, Parkinson’s disease, vascular dementia, transient ischemic attack)
5. Significant visual impairment that might interfere with the performance of investigation procedures
6. Change of treatment (i.e., psychopharmacological or psychological) 2 weeks prior to or during the study participation
7. Treatment with any neurofeedback three months prior to or during the study participation.
8. Unable or unwilling to comply with study procedures, including study prohibitions and restrictions
9. History of claustrophobia or inability to tolerate scanner environment
10. Fulfilling any of the MRI-contraindications on the standard site radiography screening questionnaire (e.g. history of surgery involving metal implants)
11. Clinically relevant structural brain abnormality as determined by prior MRI-scan
12. Planned medical treatment during the study period that might interfere with the study procedures
13. Participants deemed at significant risk of serious violence or suicide based on any of the following criteria:
  - Significant risk of committing violent acts, homicide, or suicide based on history, routine psychiatric status examination, or investigator’s judgement OR
  - Any suicide attempt in the past 3 months (i.e., actual attempt, interrupted attempt, aborted attempt) prior to screening or during the screening period OR
  - Any suicidal ideation of type 4 or 5 on the C-SSRS in the past 3 months prior to randomization or during the screening period.
14. BMI of 16.5 or lower
15. Participation in other clinical trials or observation period of competing trials, respectively
16. Previous participation in this trial
17. Pregnancy or lactation
18. Held in an institution by legal or official order
19. Legally incapacitated.

### Endpoint measures and assessments

#### Primary endpoint

The main hypothesis to be tested is whether downregulation training of amygdala activation with neurofeedback reduces the intensity of negative affect assessed before treatment as compared to immediately after treatment (i.e., at post-assessment) and whether this change is greater in the treatment group compared to the control group. The primary outcome will be assessed with a single item ‘At the moment I don’t feel well’ (deutsch: ‘Im Moment fuehle ich mich schlecht’) asking the patient to rate negative affect on a visual analogue scale ([VAS]; 0 – ‘no’, 100 – ‘very much’). Data will be sampled with the TherapyDesigner smartphone app (movisens, Karlsruhe, Germany) over 4 days, of which 2 days will be regular working days and 2 days will be weekend or holidays, over a 12-h period^1^ with hourly prompts.

#### Secondary endpoints

- Negative affect intensity assessed with EMA, group difference in change from baseline to 3-months follow-up
- Borderline Symptom Severity (Zanarini Rating Scale for BPD, ZAN-BPD [52]), group difference in change from baseline to post-assessment and to 3-months follow-up.
- Amygdala response to negative images, group difference in change from baseline to post-assessment immediately after treatment. Improvement will be quantified based on the first neurofeedback run (i.e., initial 4 view-trials [see below] of session 1) and the last neurofeedback run (i.e., last 4 view-trials of session 4).
- Amygdala self-regulation, group difference in change from baseline to post-assessment immediately after treatment. Improvement will be quantified based on the first neurofeedback run (i.e., initial 4 regulate-trials [see below] of session 1) and the last neurofeedback run (i.e., last 4 regulate-trials of session 4).
- Cost-utility analysis, i.e. the health economic impact of the intervention on quality-adjusted life years (QALY) and costs (from a societal perspective): incremental difference of costs and QALY between groups at 6-months follow-up.^2^

### Screening

An investigator will introduce the patient to neurofeedback and to the study procedures. Patients will be informed that two groups will be compared in this study, in which the relationship between the feedback display and amygdala activity will be ‘more or less immediate’ (German: ‘mehr oder weniger direkt’). Once patients have been fully informed about the study procedures and they have signed the consent form, they will be screened for eligibility. The International Personality Disorder Exam (IPDE) [26] will be used to corroborate the BPD diagnosis and a semi-structured clinician-administered interview [2] will be used to assess exclusion criteria (for a description of all assessments used in this research refer to Table 3). Demographic and medication data will be collected. If applicable, menstrual cycle will be documented to control for cycle-dependent changes in emotion regulation ability during data analysis.

**Table 3:** Description of assessments used in BrainSTEADy

**List of assessments**	
**a) Ecological Momentary Assessments (EMA)**	
Activity	Single item assessed with EMA: ‘In the moment I feel…’ (German: ‘Im Moment fuehle ich mich… ‘) to be answered on VAS (tired – awake; German: muede—wach)
Aversive tension	Single item assessemd with EMA: ‘I feel aversive inner tension right at the moment’ (German: ‘Im Moment stehe ich unter unangenehmer innerer Anspannung.‘) to be answered on VAS (not at all – very much; German: gar nicht – sehr). This item was taken from DSS-4
Emotion self-control	Mean score of two items assessed with EMA: ‘I feel overwhelmed by my feelings right in the moment’ and ‘I can handle my emotions right in the moment’ (German: ‘Im Moment fuehle ich mich von meinen Gefuehlen ueberfordert’ und ‘Im Moment habe ich meine Gefuehle im Griff’) to answer on VAS (not at all – absolutely, German: ueberhaupt nicht – voll und ganz). These are an in-house generated items used in previous research [51]
Negative experiences	Single item assessed with EMA: ‘Did you experience one or more NEGATIVE events since the last prompt? How intensive was the most meaningful one? (If you did not experience any negative event, please select „not at all “.’ (German: ‘Haben Sie seit der letzten Abfrage ein oder mehrere NEGATIVE Ereignisse erlebt? Wie intensiv war das bedeutendste? (Wenn Sie kein negatives Ereignis erlebt haben, waeählen Sie bitte „'eberhaupt nicht' aus ‘) to answer on VAS (not at all – absolutely, German: ueberhaupt nicht – voll und ganz)
Interpersonal events	Two multiple-choice items assessed with EMA (‘Since the last prompt somone has…’; German: ‘Seit der letzten Abfrage hat jemand… ‘). The first item asks for negative events (one or more options have to be selected: took advantage of me; ignored me; rejected me; did not appreciate me; did not make an effort to understand me; disappointed me; criticized me; overwhelmed me; behaved angrily or aggressively towards me; did not listen to me properly; did something else negative; did not do anything negative’; German: mich ausgenutzt; mich ignoriert; mich zurueckgewiesen; mich nicht zu schaetzen gewusst; sich nicht bemueht, mich zu verstehen; mich enttaeuscht; mich kritisiert; mich ueberfordert; sich mir gegenüber aergerlich oder aggressiv verhalten; mir nicht richtig zugehoert; etwas anderes Negatives getan; nichts Negatives getan). If the last option is ticked, the next item is shown. If one (or several) other options are ticked, the follow-up question is presented: ‘The behavior was meaningful to me (if you checked more than one behaviors, please respond with regards to the most meaningful behavior)’ (German: ‘So bedeutsam war das Verhalten fuer mich (falls Sie mehrere Verhaltensweisen angegeben haben, antworten Sie bitte in Hinsicht auf das fuer Sie bedeutsamste Verhalten)‘) to be answered on a VAS (not at all – very much; German: gar nicht – sehr) The second item asks for positive events one or more options have to be selected: supported me/helped me; took time for me; showed me affection; took an interest in me; showed me that she appreciated me; made an effort to understand me; gave me pleasure; reassured/comforted me; relieved me/took work off my hands; gave me my space; did something else positive; did nothing positive; German: mich unterstuetzt/mir geholfen; sich Zeit für mich genommen; mir Zuneigung gezeigt; sich für mich interessiert; mir gezeigt, dass sie mich schaetzt; sich bemueht, mich zu verstehen; mir eine Freude bereitet; mich beruhigt/getroestet; mich entlastet/mir Arbeit abgenommen; mir meinen Freiraum gelassen; etwas anderes Positives getan; nichts Positives getan). If one (or several) other options are ticked, a follow-up question is presented as explained above for negative events.
Negative affect intensity	Single item assessed with EMA: ‘I feel bad in the moment.’ (German: ‘Im Moment fuehle ich mich schlecht ‘) to answer on VAS (not at all – very much, German: gar nicht—sehr).
Positive affect	Single item assessed with EMA: ‘I feel good in the moment.’ (German: ‘Im Moment fuehle ich mich gut ‘) to answer on VAS (not at all – very much, German: gar nicht—sehr)
Positive experiences	Single item assessed with EMA: ‘Did you experience one or more POSITIVE events since the last prompt? How intensive was the most meaningful one? (If you did not experience any positive event, please select „not at all “.’ (German: ‘Haben Sie seit der letzten Abfrage ein oder mehrere POSITIVE Ereignisse erlebt? Wie intensiv war das bedeutendste? (Wenn Sie kein positives Ereignis erlebt haben, wählen Sie bitte „ueberhaupt nicht “ aus.‘) to answer on VAS (not at all – absolutely, German: ueberhaupt nicht – voll und ganz)
Valence	Single item assessed with EMA: ‘In the moment I feel…’ (German: ‘Im Moment fuehle ich mich… ‘) to answer on VAS (bad – good, German: unwohl – wohl)
**b) Secondary endpoints and exploratory outcomes**	
AQoL-6D	The Assessment of Quality of Live Mark 2 instrument (AQoL-6D, [37]) is a multi-attribute utility instrument that measures health-related quality of life. The instrument contains of 20 items grouped into five dimensions (illness, independent living, social relationships, physical senses and psychological wellbeing). The instrument provides weighted and unweighted age- (16 to 74 years) and gender-specific norms. Utility scores, i.e. QALYs, are elicited via time-trade off-derived (TTO) formulas. The questionnaire will be filled in at visit V1 and again six months after the last neurofeedback session in FUP2.
BDI-II	Depressivity is measured with the Beck Depression Inventory (BDI-II [15]), which is a widely established 21-items self-report questionnaire. Items are rated on a scale from 0 to 4, a total score is received by averaging across all items. Higher values indicate higher depressivity
BSL-23	The Borderline Symptom List short version (BSL-23 [48]) is an established self-rating questionnaire to assess Borderline symptom severity within a 1-week time frame. It is composed of 23 items. Each item is rated on a scale from 0–4 (0 = not at all to 4 = very strong/several times a day). Total score is received by averaging across the 23 items. The scale will be administered at visits V1, V5 and FUP1.
CGI	The Clinical Global Impression (CGI) rating scale is a measure of symptom severity, treatment response and of the efficacy of treatments in studies of patients with mental disorders. The CGI-S will be used at V1 to evaluate severity, and the CGI-I will be used at V5 to evaluate improvement.
Concluding questionnaire	An in-house developed, single-page self-report questionnaire asking the patient to rate their experience with the treatment after V5 (see Supplement).
DERS	The Difficulties in Emotion Regulation Scale [12], is a 36-item self-report measure. Items are rated on a scale of 1 (almost never) to five (almost always). Higher scores indicate more difficulty of emotion regulation.
DES	In order to better characterize the sample with respect to dissociation, we will assess trait dissociation at baseline based on the most widely used assessment instrument, i.e. the Dissociative Experiences Scale (DES) [5] (28 items).
DSS-4	To investigate whether state dissociation during the fMRI-neurofeedback might be detrimental to the effect of the treatment, a brief assessment of state dissociation (4 items on dissociation + 1 item asking for aversive tension) following each neurofeedback-session will be administered.
DSS-acute	To investigate whether fMRI-neurofeedback is improving patients’ dissociation a comprehensive assessment of state dissociation, the DSS-acute (C. E. [43]) (22 items) will be assessed.
FIMPsy	The “Fragebogen zur Inanspruchnahme medizinischer und nicht medizinischer Versorgungsleistungen bei psychischen Erkrankungen” (FIMPsy [13]) assesses health care resource use (including outpatient and inpatient medical care, intake of medication, informal care, psychiatric counselling, psychosocial care, social participation, vocational (re-)integration,) in patients with mental disorders over the previous six months. The FIMPsy facilitates health economic evaluations by collecting type, frequency or duration of health care utilization. For this research an additional chapter was added to investigate digital health applications.
MSQ-NF	Mental strategies used by participants for amygdala downregulation during the neurofeedback training will be assessed using Mental Strategies Questionnaire for NeuroFeedback (MSQ-NF, [28]). Patients will determine their own mental strategy for every regulation block and fill out the questionnaire at the end of every neurofeedback visit (V2b, V3, V4, V5a) for every distinct mental strategy separately. MSQ-NF allows to characterize strategies across dimensions, and identify the features of successful mental strategies.
PCL	The Paranoia Checklist ([24], PCL; 18 items) measuring the extent of paranoid symptoms, will be assessed for prediction of potential non-response to therapy.
RSQ	The Relationship Scales Questionnaire [42] was developed as a continuous measure of adult attachment. The RSQ contains 30 short statements on a 5-point scale ranging from '"not at all like me' "to '"very much like me', participants rate the extent to which each statement best describes their characteristic style in close relationships.
SCL-27	The Symptom Checklist (SCL-27, [14]) is designed to screen for psychological complaints in patients with leading physical symptoms. The subscales depressive, dysthymic, vegetative, agoraphobic, sociophobic symptoms and symptoms of mistrust are formed, as well as a global severity index (GSI-27).
Survey of NF transfer	Short questionnaire to ask patients whether they used self-regulation strategies learned during neurofeedback in daily life and how effective they were (see Supplement).
SCID	The Structured Clinical Interview for DSM-5 is a semi-structured clinician-administered interview for the assessment of psychiatric diagnosis according to the Diagnostic and Statistical Manual of Mental Disorders Version 5 [2].
WPAI-GH	The General Health (GH) version of the Work Productivity and Activity Impairment Questionnaire (WPAI-GH, [36]) is a questionnaire designed to assess presenteeism and absenteeism due to health problems and work or activity impairments. The WPAI:GH consists of six questions, which elicit the amount of productivity loss to society over the past seven days. The questionnaire will be filled in at visit V1 and again six months after the last neurofeedback session at FUP2.
ZAN-BPD	Symptom severity will be assessed with the Zanarini Rating Scale for BPD ([52]; ZAN-BPD). ZAN-BPD is a structured clinical interview administered by trained staff, who are blinded with respect to group allocation. The questions are adapted from the Diagnostic Interview for DSM-5 Personality Disorder to reflect a 1-week time frame. Each criterion is rated on a scale from 0–4, yielding a total score of 0 to 36.
**c) Other assessments (process-related, anamnestic)**	
AEs	AEs will be asked for at each contact between the responsible investigator and the subject. Furthermore, new pathological and clinically relevant findings or aggravation of pre-existing symptoms in examinations will be documented as AEs. AEs will be reported with subject ID, start and end date, description, grading, seriousness, relatedness, action taken and outcome.
Blind check	An in-house developed questionnaire. Subjects as well as responsible investigators are asked about their belief in what experimental condition they were randomized (i.e., amygdala-feedback or sham-feedback). There is a version for patients and a version for trial staff (see Supplement).
C-SSRS	The C-SSRS (https://cssrs.columbia.edu/) is a semi-structured, investigator-rated interview, developed by clinical experts in cooperation with the FDA, assessing both suicidal behavior and suicidal ideation. It does not give a global score but provides some categorical and some severity information specifically for behavior and ideation.
IPDE	The International Personality Disorder Examination (IPDE, [26]) is a clinician-administered semi-structured interview used to assess personality disorders as defined in the DSM-5 and ICD-11. The BPD subsection will be used to assess for the presence of severity of symptoms related to BPD. It will be administered during the diagnostic interview.
Medical history and underlying disease history	Clinically significant diseases, surgeries, previous medical procedures, smoking history, use of alcohol and/or drug abuse, reproductive status, and all medications (e.g., prescription drugs, over-the-counter drugs, herbal or homeopathic remedies, and nutritional supplements) taken by the subject within 7 d prior to start of investigational treatment.

### Baseline assessment

After the screening visit, patients will complete their first EMA assessment, while following regular daily activities. The app will issue an alarm 12 times a day on four consecutive days, and will present 11 items addressing emotional state, experiences and interpersonal events (see Table 3). Additionally, patients will complete (electronic) psychometric questionnaires and assessments of quality of life. Self-report data will be collected online via a browser-based interface programmed in MARVIN software (EventIQ, Hamburg, Germany). Measures of psychopathology will be assessed on-site by trained staff, and patients will receive their first MRI-measurement including an anatomical scan, Diffusion Tensor Imaging (DTI), and neurofeedback.

### Randomization and adverse events assessment

When patients responded to a minimum of 50% of EMA prompts, they will be randomized to the verum or the control condition before they are going to receive their first MRI-measurement. The operator of the neurofeedback software Turbo-BrainVoyager MED Borderline-Personality Disorder (TBV MED BPD, Brain Innovation, Maastricht, Netherlands) will enter a patient ID in the beginning of neurofeedback training, and an algorithm will automatically assign the subject either to the verum- vs. control-condition. This assignment will be based on a randomization list created by an independent statistician (CRO), which was implemented into the software by the software developer (who is not the sponsor of the trial), where it will be hidden from the operator. Stratified permuted block randomization with trial site as a stratum was applied. Within each stratum, block sizes are variable to protect concealed assignment. When unblinding of a patient’s group assignment during the trial will become necessary, the sponsor representative can send a request to the CRO. Following randomization, adverse events will be assessed throughout the study on each visit. We will interrogate both, adverse events happening between visits and adverse events happening specifically during the MRI scans.

### Neurofeedback

The initial three neurofeedback sessions will be administered within 14 days, with 2–7 days in-between consecutive sessions. Session 4, which we also call a ‘booster’-session, will be administered four weeks after session 3. In the interim period between sessions 3 and 4, subjects will receive a weekly electronic questionnaire asking them how often they were using emotion regulation strategies in daily life and how effective those strategies were (cf. Tables 2 and 3).

#### Session preparations

In the beginning of the first MR visit, the patient will receive detailed verbal instructions about neurofeedback and mental strategies (see Supplement). Then, the subject will enter the MR-suite. During scanning, subjects will see a computer monitor at the back of the MR-bore via a mirror mounted on the head coil. Before each neurofeedback run, the subject will name two to four mental strategies. The experimenter will enter the strategies into a visual interface during subject registration with TBV MED BPD before each neurofeedback run. The strategies will be presented to the subject before each neurofeedback cycle and the subject has to decide, which strategy they want to practice in the upcoming cycle.

#### MRI acquisition

**Fig. 3:**
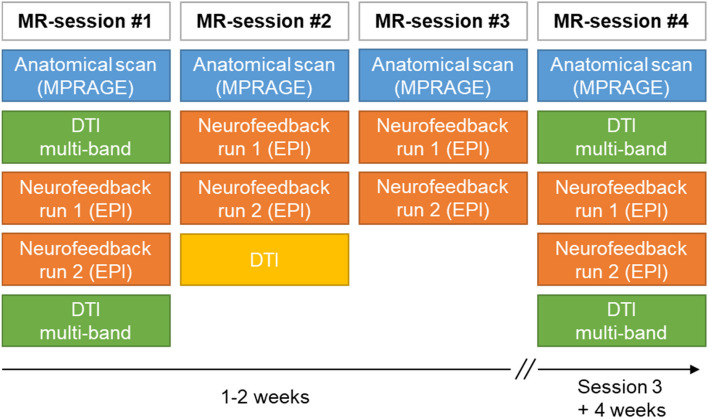
Schedule of Magnetic Resonance (MR) sessions. DTI = Diffusion Tensor Imaging, EPI = Echo-planar imaging

MR data will be acquired at a 3 T Prisma/PrismaFit scanner (Siemens Healthineers, Erlangen, Germany) with 64-channel headcoil. First, an anatomical 3D T1-weighted scan will be acquired (Magnetization Prepared Rapid Acquisition Gradient Echo [MPRAGE] sequence, TE = 3.03 ms, TR = 2.3 s, 192 slices and field of view (FOV) = 256 × 256x192 mm). Then, the measurement schedule will differ between the four MR visits. At MR sessions 1 and 4, we will run multi-band DTI-scans before and after neurofeedback to assess fast structural changes of the brain (Fig. 3).

Following the MPRAGE sequence, and both immediately before and immediately after the neurofeedback runs, four DTI acquisitions will be performed. Two scans will use an anterior-to-posterior (AP) phase-encoding direction and two will use a posterior-to-anterior (PA) direction, alternating in the sequence AP, PA, AP, PA. The imaging parameters for each acquisition are TE = 54 ms, TR = 2500 ms, FOV = 220 mm, matrix size = 88 × 88, flip angle = 90°, GRAPPA acceleration factor = 2, 35 diffusion directions, and b_1_ = 1000 s/mm^2^. Each scan will consist of 56 slices tilted 16° toward the posterior commissure (PC) from the anterior–posterior commissure (AC–PC) plane. At MR session 2, following the neurofeedback runs, we will perform a structural DTI sequence with the following imaging parameters: TE = 88 ms, TR = 3900 ms, FOV = 222 mm, matrix size = 148 × 148, flip angle = 90°, SMS factor = 2, phase-encoding direction AP, 65 diffusion directions and three q-shells (including b0, **b**_**1**_ = 1000 s/mm2, and **b**_**2**_ = 2000s/mm2). Each scan will consist of 60 slices tilted 16° toward PC from the AC–PC plane. Subsequently, we will acquire a DTI sequence for distortion correction using identical parameters, except with a PA phase-encoding direction, 12 diffusion directions, and two q-shells (including b0 and **b**_**1**_ = 500 s/mm2). This second sequence likewise consists of 60 slices tilted 16° toward the PC from the AC–PC plane.

Two neurofeedback runs with 431 volumes each will be conducted. Functional images of the BOLD contrast will be acquired with gradient echo T2* weighted echo-planarimaging sequence (TE = 30 ms, TR = 2 s, FOV = 192 mm, matrix size = 64 × 64, flip angle = 80°, GRAPPA acceleration factor = 2). Each volume consists of 36 slices tilted 16° toward the PC from the AC–PC plane, with thickness 3 mm and slice gap 1 mm. Participants' heads will be lightly restrained using soft pads to prevent head movement. A strip of medical tape will be fixed to the patients’ forehead to reduce involuntary movement as described by Krause et al. [20].

#### Real-time fMRI setup

The MR scanner will export reconstructed images via TCP/IP to the neurofeedback computer running TBV MED BPD. The software will process the data and will provide graphical output via a computer monitor to the subject in the scanner. The subject will use a keypad to respond to rating scales during the MRI-session.

#### Real-time fMRI data processing and feedback

**Fig. 4:**
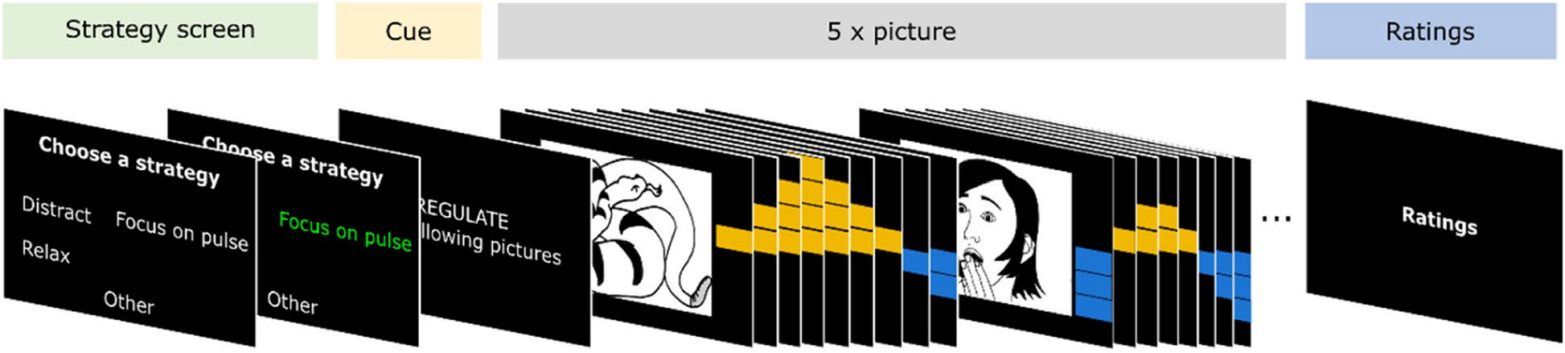
Neurofeedback interface: Stimulus structure of regulate-trial. The trial starts with the strategy screen where the patient chooses a mental strategy that should be used for self-regulation for the upcoming series of pictures. Strategy names shown here are illustrative and will be adjusted to the individual preferences of the patient upfront training as described in the main text of this article. The subject has to select a strategy and confirm, indicated by green font colour. Following the instruction cue, five pictures are shown with a visual analogue scale (VAS) indicating real-time brain activation. Finally, arousal and self-regulation success ratings are done. Timing information can be obtained from Fig. 5

TBV MED BPD will be used for real-time data processing and stimulus presentation. The right amygdala region-of-interest (ROI) produced with the Harvard–Oxford brain atlas with a probability threshold of 25% will be used to define the neurofeedback target region. At the beginning of the session, the anatomical image will be normalized to Montreal Neurological Institute (MNI) standard space [19]. Normalization vectors will be used to transform the ROI into each subject’s functional native space. A short functional scan (5 volumes) will be processed by TBV MED BPD to calibrate the software and verify proper alignment before the start of the neurofeedback paradigm. The fMRI data will be preprocessed in real-time using 3D motion correction to the first volume of the short functional scan (for each neurofeedback run). Additionally spatial smoothing with a 3-mm FWHM kernel will be performed. The ROI time course will then be further preprocessed using a real-time General Linear Model (GLM) based detrending routine including 6 motion parameters (translation x,y,z and rotation x,y,z) as well as a linear trend confound predictor. The resulting neurofeedback signal will then be normalized to Percent Signal Change (PSC) values based on the estimated beta amplitudes in the real-time GLM. Feedback will be displayed via VAS. The VAS will be separated in an upper and lower part, representing positive and negative signal change from baseline. The mean value of the VAS will be set to an arbitrary baseline as described below. An increasing number of yellow bars (max. 4 bars) indicates an increase in positive activation, while an increasing number of blue bars (max. 4 bars) means that the signal is decreasing (Fig. 4). In the outset of a neurofeedback run, the mean and the range of the VAS will be configured to Baseline = 0.87 PSC, Min = −0.75 PSC, Max = 2.00 PSC. These values will be adapted dynamically based on the subject’s performance before a new cycle begins. The maximum value will be based on the amygdala peak response to pictures of the previous view- and regulate-trials (AmyPeak). AmyPeak is determined as the average value between 6–10 s post-stimulus onset. The new maximum value is calculated by averaging AmyPeak with the maximum value from the previous cycle: Max_new_ = [AmyPeak + Max]/2. In case of extreme values (Max_new_ < 1.00, Max_new_ > 2.00), the lower or upper cut-off values will be used instead (i.e., Max_w_ 1.00 and 2.00, respectively). A similar approach will be taken to dynamically adjust the minimum of the VAS. The trough of the amygdala response (AmyTrough) will be determined as the average value during the arousal ratings.^3^ The new minimum is Min_new_ = [AmyTrough + Min]/2. If Min_new_ is below −1.50 or above −0.50 it will be fixed at −1.50 or −0.50, respectively. The new Baseline will be set to the midpoint between the updated maximum and minimum (Baseline = [Max + Min]/2).

In the verum-condition, the feedback will be derived from real-time fMRI data from the subject’s right amygdala. In the control-condition, the feedback signal of a subject from the verum-group will be replayed instead of the subject’s own signal. If a subject is randomized to the control-condition and no suitable verum-condition feedback data is yet available, pre-recorded data will be used.

#### Stimulus presentation

A regulate-trial will begin with presentation of a strategy-choice screen. Three to five strategy options will be displayed for six sec. The headline of the strategy screen will read ‘Choose a strategy’ (German: ‘Waehle eine Strategie aus’), followed by a list of two to four mental strategies including an ‘other’ option. The subject uses a keypad to move the cursor and to confirm their choice. For the next four sec the text ‘REGULATE the following pictures’ (German: ‘REGULIERE die folgenden Bilder’) will be displayed (i.e., the cue-phase). Then, five pictures will be displayed, each for 18 s. Next, the subject will rate their arousal on a nine-level VAS (SAMmy scale, 1 = no arousal, 9 = very high arousal, Online Supplement) and their subjective brain self-regulation success (‘Could you regulate your brain activation’, German: ‘Konntest du deine Gehirnaktivierung regulieren?’) on a nine -level VAS (1 = not at all, 9 = very good). Subjects will have eight sec to complete each rating (Fig. 4).

A ‘view’-trial will start with the instruction ‘VIEW the following pictures’ (German: ‘BETRACHTE die folgenden Bilder’), which will be presented for four sec on the screen. Two pictures will be presented, each for 18 sec. Then, subjects will rate their arousal.

**Fig. 5:**
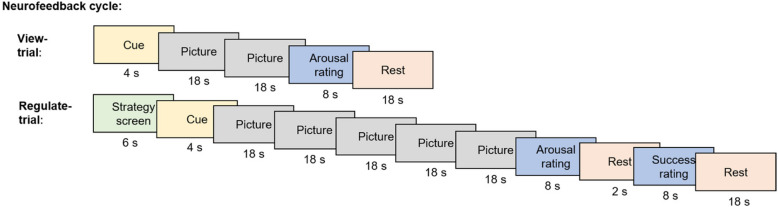
Stimulus timing of neurofeedback experiment. A neurofeedback cycle is composed of a view-trial and a regulate-trial. During ‘Cue’ condition participants are instructed about the next trial type (i.e., view, regulate). During ‘Rest’ condition a white fixation cross is displayed on black background. A full neurofeedback training session has eight cycles. The session is separated in two runs (four cycles per run)

We call a series of a ‘view’-trial and a ‘regulate’-trial a cycle (Fig. 5). During each session, subjects will complete two neurofeedback runs with four cycles per run. Scanning will stop after each run where the neurofeedback trainer can talk to the subject.

#### Stimulus material

The pictures displayed during neurofeedback were selected from several published databases [6, 7, 21, 23, 30, 47], based on rated valence and arousal provided in the original publications. Ratings of images that were collected with a rating scale other than the widely used one to nine VAS (1 = negative valence/low arousal, 9 = positive valence/high arousal; i.e., GAPED, OASIS) were recalculated in order to receive comparable ratings of each image. Images of negative valence (rating below five) and moderate or high arousal (rating above 3.5) were selected. The arousal level of images was balanced between neurofeedback runs, cycles and conditions with three steps: First, images were assigned to runs and equal distribution of arousal and valence across runs was assessed using ANOVA (*p* > 0.9). Second, a similar procedure was used to generate balanced arousal and valence levels across cycles within each run. Finally, images were shuffled between the view- and the regulate-condition to achieve a close match of arousal levels. A list of the original image codes used in this research can be found in Supplementary Table S10.

#### Description and transfer of mental strategies

After completion of each session, the patient will fill in the Mental State Questionnaire for Neurofeedback (MSQ-NF, [28]) outside the MR suite. The MSQ-NF systematically assesses mental strategies used during neurofeedback to better understand their impact on learning outcomes and to inform protocol refinement. It is hierarchically structured into general meta-categories comprising 45 features. These features cover key mental modalities, contents, and dimensions from affective and cognitive psychology, including exteroception, interoception, affect (e.g., emotions, arousal, valence), imagery, memory, language, motor skills, arithmetic, and social aspects. Patients will complete one version of the MSQ-NF for each cycle. After the third neurofeedback session, the trainer and the patient will together create a list of four to six mental strategies, which the patient has to practice in everyday life during the four-week interim period between session 3 and session 4. During this study phase, patients will receive a weekly electronic (in-house developed) questionnaire called ‘Survey of Neurofeedback transfer’ with questions asking about the frequency of strategy use and perceived success (see Supplement).

### Discontinuation of participation

Reasons leading to discontinuation of included particpants in this trial include:

- Patient withdraws consent to participate;
- Any exclusion criterion for MR scanning apply;
- Positive pregnancy test;
- Incomplete primary endpoint assessment, i.e., > 50% EMA alarms were missed;
- Patient misses first neurofeedback session (V2), unavailability of patient to reschedule session within 14 days of primary endpoint assessment at baseline;
- Patient misses last neurofeedback session (V5a), unavailability of patient to reschedule session within 7 weeks after neurofeedback session 3 (V4);
- Patient is unavailable for primary endpoint post-assessment within 21 days of last neurofeedback session (V5a);
- Patient does not comply with study procedures.

Patients who discontinue to receive the treatment should be motivated by trial staff to receive post- and/or follow-up assessments.

### Post-assessment and follow-up

In the week after neurofeedback session 4, patients will complete EMA again. Following completion of EMA, subjects will fill in (electronic) psychometric questionnaires and undergo on-site ZAN-BPD assessment (i.e., post-assessment of endpoint measures). Three months later, they will complete the first round of follow-up assessments (including EMA), and three months later, the 6-months follow-up will be done (c.f. Table 2).

### Statistical analysis

#### Primary endpoint analysis

The hypothesis will be tested at the one-sided overall significance level α of 0.025. The primary endpoint will be analyzed on the full analysis set in stage 1 after 82 participants (n_1_ = 41 per group) have reached this endpoint according to the Intent-To-Treat (ITT) principle. The effect of the intervention on the primary endpoint will be tested using a time*condition interaction of a mixed linear model with the mean score of negative affect as the dependent variable, time as within-subject factor, condition (verum vs. control) as between-subject factor, trial site as a random factor, and sex as a cofactor. If the p-value pertaining to the primary outcome in stage 1 (p_1_) exceeds the critical threshold of α_0_ = 0.3000, the trial will be stopped for futility, i.e., without rejection of the null hypothesis. If p_1_ ≤ α_1_ (with α_1_ = 0.0131 according to Bauer & Köhne, [1], p. 1,031) the null hypothesis (H0) can be rejected at stage 1 and the trial will be terminated. If α_1_ < p_1_ ≤ α_0_, the trial will be continued (stage 2) with an additional sample size of n_2_ = 41 patients per group. If the trial enters stage 2, the null hypothesis is rejected at the final analysis if the product of the stage-wise p-values p_1_p_2_ falls below the critical boundary of c_α_ = 0.0038.

#### Power analysis

Power calculations were carried out to determine the sample size needed for achieving an overall power of 1-β ≥ 0.90 for the primary endpoint. This power analysis is based on the improvements (i.e. pre-post differences of the mean negative affect) and the pooled standard deviation of these improvements as observed in our pilot data [51]. Mean improvements were 0.37 ± 0.52 in the group with amygdala-NF and 0.03 ± 0.44 in the control group. This translates to a between-group difference in the improvements of 0.34 and a pooled standard deviation of 0.46, which corresponds to a standardized mean difference (SMD) of δ_1_ = 0.74. Despite the lack of established minimally relevant difference, SMDs above 0.50 are likely highly relevant because negative affect assessed in real life conditions is crucial for individuals with BPD and closely relates to dysfunctional and self-harming behaviors. The utility of an add-on intervention that can be combined with traditional treatment is particularly high. As suggested by the International Committee of Harmonization (ICH) E9 guideline, sensitivity analyses were considered when determining the sample sizes n_1_ and n_2_ (i.e. the n’s per group at stages 1 and 2 of the sequential trial). To this end, the following scenarios were investigated: Scenario (S1) based on the point estimates according to the pilot data analyses, i.e. an absolute group difference (AGD) of 0.34 in the improvement of the mean negative affect and a pre-to-post correlation (r = 0.82) required for specifying the correlation matrix in the power analysis were complemented by scenarios (Sa-Sc) based on more conservative, albeit realistic values for r and the AGD. For correlation r, the lower end of the 95% confidence interval (i.e. r = 0.73) was considered, while sensitivity analyses alternatively considered an AGD of 0.23 (instead of 0.34), which corresponds to a medium SMD of 0.50, which is both plausible according to the pilot data and still presents a clinically meaningful difference. Furthermore, as suggested by Pilz et al. [35], we set a constraint for the minimal conditional power of 0.70 for stage 2 and have split n1 and n2 to minimize the expected total sample size. These conditions were required for all scenarios considered under H1 (i.e. S1, Sa-Sc). Accordingly, n_1_ = 41 plus (possibly) n_2_ = 41 participants per group are needed to achieve an overall power of 90% in all of these scenarios. Accordingly, the maximum total sample size is 164.

#### Cost utility analysis

We measure utilization of healthcare services, medication, remedies (direct cost), loss of productivity (indirect costs) and estimate costs of neurofeedback treatment (micro-costing) and measure utilities (QALY) to calculate incremental cost-effectiveness ratios (ICER) and the net-monetary benefit (NMB).

We measure direct costs with the questionnaire on the resource use of medical and non-medical care services for mental illnesses (FIMPsy, c.f. Table 3), indirect cost (i.e. loss of productivity) with the Work Productivity and Activity Impairment Questionnaire: General Health (WPAI:GH) and perform micro-costing of the intervention itself with the help of a costing interview with specialists according to the intervention. We measure QALYs with a preference-weighted generic instrument, the AQoL-6D.

We will calculate incremental cost-effectiveness ratios (ICER) and the net-monetary benefit (NMB) of the neurofeedback treatment. To confirm the uncertainty around the ICER, we perform non-parametric bootstrapping with 5,000 replications and 95% confidence intervals presented in cost-effectiveness diagrams. We present willingness-to-pay thresholds as a function of the likelihood of cost-effectiveness according to specific monetary values in cost-effectiveness acceptability curves [16].

#### fMRI data analysis

fMRI data will be preprocessed offline using fMRIPrep [9]. Individual data will be screened for exaggerate movement based on Framewise Displacement (FD). Measurements with FD > 3 mm will be removed from the analysis. A mass-univariate GLM approach will be used at the subject level for modeling of task-related effects. The BOLD response from the amygdala to pictures (i.e., mean signal from amygdala voxels) will be modeled for the regulate condition and the view condition separately. The right amygdala-ROI will be defined with the same anatomical amygdala mask that was used for neurofeedback. The effect of the intervention on imaging endpoints will be tested using a time*condition interaction of a mixed linear model with the height of the amygdala response as the dependent variable, time as within-subject factor, condition (verum vs. control) as between-subject factor, trial site as a random factor, and sex as a cofactor.

### Patient involvement

A patient board consisting of three volunteers with lived experiences supported the improvement of trial methods in terms of utility and relevance of this study for individuals suffering from BPD. Two on-site board meetings where conducted during the planning phase. The board members reviewed study materials including the informed consent form and instructions for readability and clarity. They tested the neurofeedback training and contributed to the optimization of the graphical display of the visual interface. Additionally, board members contributed to the selection process of items for EMA.

### Data management

A Clinical Research Organization (CRO, i.e., KKS Heidelberg) will provide and manage the database for electronic data entry and for electronic patient reported outcomes. The recruiting trial site will collect and keep personal information of potential and enrolled trial participants with exclusive access for authorized personnel. Patient data will be pseudonymized before it will be shared with the sponsor. Adverse events, device deficiency and protocol violations will be documented following established routines. EMA and MRI data will be managed at the Central Institute of Mental Health, Mannheim. The CRO will provide regular monitoring of data quality and study conduct at the study centers. Personal information of subjects is saved separately from other study data at the study centers. Documentation, collection, storage, transfer and archiving of data will comply with EU Medical Device Regulations, applicable German law (Medizinprodukte Durchfuehrungsgesetz), DIN EN ISO:14155 (covering GCP), and General Data Protection Regulation. An independent statistician at the CRO will keep randomization information (i.e., randomization of subjects to verum vs. control) secret until the study ends. Additionally, the statistician will be responsible for communicating the decision to continue or to stop the trial following interim analysis. More details on data management, safety, and other aspects of this trial can be obtained from the Clinical Investigation Plan (CIP), which can be downloaded from the ClinicalTrials.org website.

Data will be cleaned, prepared for analysis, analyzed, and interpreted at the Central Institute of Mental Health, Mannheim, in collaboration with the scientific partners of the trial located at the study centers in Freiburg, Giessen and Tuebingen. They will write the final report and publish the results of the trial in a peer-reviewed scientific journal. Further details on publication policy can be obtained from the CIP. The funding agency (DFG) was not involved in the conceptualization of the trial and will not involve in any activity mentioned above.

## Discussion

### Discussion of the current literature

A systematic literature search was done to evaluate safety, clinical performance and clinical benefit of neurofeedback. In conclusion, according to the best available evidence fMRI-neurofeedback seems to be safe for both healthy and for several clinical populations. Further clinical investigations should systematically assess and report the presence or absence of undesirable side-effects or adverse events.

With the current evidence, it can be concluded that some patients with BPD can achieve downregulation of amygdala activation using neurofeedback. To date it is unknown whether regulation performance can be improved further with an increasing number of neurofeedback sessions (the studies used three to four sessions). It can be expected, that safety and clinical performance can be evaluated in more depth following publication of more clinical trials.

The existing neurofeedback studies found improvements in emotion dysregulation and BPD symptoms. Limitations arise from small sample sizes and the lack of control groups.

However, available data on the potential benefit of fMRI-neurofeedback for BPD is limited. There is an urgent need for more research to corroborate findings with well-powered, controlled trials.

### The BrainSTEADy trial

BrainSTEADy investigates an assumed therapeutic mechanism, i.e., that self-regulation of brain reactivity to emotional cues can improve BPD symptoms. To the best of our knowledge, this study is the first placebo-controlled neurofeedback RCT done in BPD.

In the following we will discuss common challenges in RCTs conducted with neurofeedback and how we addressed them in BrainSTEADy, i.e., choosing a suitable control group, blinding, and ecological validity of outcomes. We assume that the specific treatment effect of neurofeedback results from a change in neural patterns targeted by the treatment, i.e., from the attenuation of amygdala hyper-reactivity. However, researchers suspect neurofeedback to invoke a number of unspecific effects that go beyond the typical psychosocial effects considered in pharmacological treatment research [27]. Based on the premise that reward is the primary driver of neurofeedback learning [29], we chose a yoked-feedback control group to match as closely as possible the dosage of reward received by the experimental group [40]. Unbeknownst to the subject (and to the investigator), the feedback received by subjects in the control group is not from their own brain, but is played back from a previous subject in the verum group. The verum group differs from the control group in the contingency of the feedback with amygdala activation, while other aspects of the procedure remain constant across groups. Thus, yoked-feedback can be considered a suitable placebo control-treatment in neurofeedback research. In contrast to pharmacological research, where the effects of interest are supposed to result from the molecular properties of the compound, subjects are actively trying to regulate their brain activation in neurofeedback. Thus, yoked feedback is not only without contingent feedback, just as the control treatment in pharmacological research is lacking an active ingredient, it has even been suggested that yoked feedback can invoke detrimental effects. For instance, yoked feedback could foster the experience of failure to self-regulate, which could frustrate subjects and could potentially aggravate symptoms such as depression or negative self-esteem. Although such concerns have been raised in the literature (e.g. discussed in Gerchen et al. [10]), we are not aware of empirical evidence that yoked-feedback can have such undesired effects. With the BrainSTEADy trial, we are aiming to fill this gap in knowledge by closely monitoring and following up on adverse events.

A critical aspect of placebo-control is blinding. A comparator that is lacking characteristic features of the verum-condition might make patients (and investigators) suspicious that they are receiving (or administering) the control treatment. Thus, it is paramount to prevent unintended unblinding of patients and trial staff and to assess whether the blinding was maintained. To accomplish this, we will obtain informed consent without providing explicitly the details of the control procedure to patients during the screening visit. Second, TBV MED BPD has been programmed with additional features to conceal group allocation from the operating staff. The randomization list will be shared with the biostatistician for data analysis only after the last patient was assessed. A planned interim analysis will be done when half of the full sample has been recruited. To maintain blinding, an independent statistician from a CRO will conduct the interim analysis and will inform the sponsor whether to continue or to stop recruitment based on the analysis of the primary endpoint. The algorithm underlying the decision has been defined a-priori. Blinding of patients and trial staff will be monitored with an in-house developed questionnaire administered after the post-assessment; both subjects and study personnel will indicate whether they believe they were allocated to the amygdala-feedback or the yoked-feedback group using a forced-choice approach (Blind check-questionnaire, Online Supplement, c.f. Table 2).

This trial will implement hourly assessments of affective state, so-called ecological momentary assessments (EMA), while patients go about their regular daily activities. In contrast, most established clinical assessments use a retrospective approach, which is prone to memory bias and does not capture well the highly volatile moment-to-moment emotional changes that are characteristic for BPD. The combination of both EMA and standard retrospective assessments in this trial will enhance the ecological validity of the results [31].

Besides providing evidence on the efficacy of fMRI neurofeedback, this research will address further research questions of importance for neurofeedback and treatment. Using Diffusion Tensor Imaging (DTI), we are aiming to delineate neural plasticity induced by neurofeedback. A recent neurofeedback study [38] on regulating the activity of sensorimotor cortices showed rapid effects on white matter structure measured with DTI. In a sham-controlled neurofeedback study, healthy individuals performed a motor imagery task (1 h) and showed increased fractional anisotropy in sensorimotor segments of corpus callosum. DTI allows tracking of learning-related neuroplasticity [4]. Previous work has indicated that functional amygdala-prefrontal connectivity can be altered by neurofeedback training [34]. However, it remains unknown what neuroplastic effects neurofeedback training has on the target structure (amygdala) itself, as well as on structures that exert a regulatory influence on the amygdala (e.g. prefrontal regions and hippocampus). With this research, we are aiming to bring light to this issue. Furthermore, we will explore whether neurofeedback could be beneficial for patients who may respond poorly, or may even drop out early, from established treatments. We will focus on a few variables that were previously found to relate to poor response such as attachment patterns, paranoia [49], baseline levels of distress and non-acceptance of emotions [22] and dissociation [17, 18, 41].

We would like to highlight the systematic approach to address the mental strategies participants use to regulate their brain activity. This approach involves documenting the trial-by-trial mental strategies used during training sessions and characterizing these strategies in feature space with the MSQ-NF questionnaire that was recently introduced for neurofeedback research [28]. Lastly, usage and effectiveness of mental strategies during everyday life will be assessed with weekly questionnaires. This protocol may elucidate whether there are common features of mental strategies that are effective for amygdala regulation and that are suitable for transfer to daily life.

To improve the quality of the research and to warrant patient safety, we established both a Data Safety Monitoring Board (DSMB) and a patient advisory board. The DSMB consists of three experts in clinical research, BPD and neurofeedback, indicating no competing interests. The DSMB will monitor safety parameters and the recruitment progress. The DSMB charter can be obtained from the sponsor upon reasonable request. The three members of the patient board provided valuable insights from the perspective of patients and study participants during the different phases of planning and will be involved in the interpretation of results.

Access to effective BPD treatment is limited. If shown to be effective, fMRI-neurofeedback could be offered to patients who are on a waiting list for a psychotherapy. FMRI-neurofeedback could also be integrated in the patient’s treatment plan to support the training of emotion regulation skills or to reduce the general stress level of patients. As emotion regulation training is a major building block of BPD-specific treatment programs such as Dialectical Behavior Therapy, fMRI-neurofeedback might particularly benefit patients with dissociative tendencies or with difficulty engaging in therapeutic lessons. If corroborated by research, fMRI-neurofeedback could become a treatment option for patients who have not benefited from previous treatments.

## Supplementary Information

Supplementary Material 1

Supplementary Material 2

## Data Availability

Materials can be found in the Online Supplement.

## Abbreviations

AGD: Absolute Group Difference
AC: Anterior Commissure
AP: Anterior-to-Posterior
BOLD: Blood Oxygenation Level Dependent
BPD: Borderline-Personality Disorder
CIP: Clinical Investigation Plan
CRO: Clinical Research Organization
EEG: Electroencephalography
FOV: Field Of View
FD: Framewise Displacement
GLM: General Linear Model
GCP: Good Clinical Practice
DSMB: Data Safety Monitoring Board
DTI: Diffusion Tensor Imaging
EMA: Ecological Momentary Assessment
ICER: Incremental Cost-Effectiveness Ratios
MDD: Major Depressive Disorder
MPRAGE: Magnetization Prepared Rapid Acquisition Gradient Echo
MRI: Magnetic Resonance Imaging
NMB: Net-Monetary Benefit
PA: Posterior-to-Anterior
PC: Posterior Commissure
PSC: Percent Signal Change
QALY: Quality Adjusted Life Years
RCT: Randomized Controlled Trial
ROI: Regions-Of-Interest
SMD: Standardized Mean Difference
TBV MED BPD: Turbo-BrainVoyager MED Borderline-Personality Disorder
TE: Echo time
TR: Repetition time
VAS: Visual Analogue Scale

## Footnotes

1 The subject can change the assessment period by selecting one of three options: a) 8am-8 pm, b) 9am-9 pm, c) 10am-10 pm.

2 6-months follow-up was introduced for the cost-effectiveness endpoint, because a 6-months post-treatment period as compared to a 3-months post-treatment period improves the validity of the endpoint assessment.

3 The subject rates their arousal in the end of each regulate-trial and in the end of each view-trial. The arousal-rating period of view-trials will only be considered when values are below the current minimum. This approach is based on the assumption that the amygdala may respond more strongly in view-trials as compared to regulate-trials, which may result in an elevated amygdala baseline signal in the end of view-trials as compared to regulate trials. In such scenario, re-setting the minimum based on view-trials would rather not represent well the expected signal range during regulate-trials.
