# Supplementary material for: A randomised controlled trial of amygdala fMRI-neurofeedback versus sham-feedback in borderline-personality disorder – systematic literature review and introduction to the BrainSTEADy trial
Source: BMC Psychiatry. 2025 Jul 8;25:687. doi: 10.1186/s12888-025-07000-1 (PMC12235889; doi:10.1186/s12888-025-07000-1)

**A randomized controlled trial of amygdala fMRI-neurofeedback versus sham-feedback in Borderline-Personality Disorder – Systematic literature review and introduction to the BrainSTEADy trial**

Online Supplement

List of content

[2 Tables 3](#_Toc200538017)

[2.1 Table S1. Trial Synopsis 3](#_Toc200538018)

[2.2 Table S2. Search strategy and search terms with results 7](#_Toc200538019)

[2.3 Table S3. Publications screened on abstract level 10](#_Toc200538020)

[2.4 Table S4. Publication type of potentially relevant publications 10](#_Toc200538021)

[2.5 Table S5. Description of Clinical Population in Potentially Relevant Publications 11](#_Toc200538022)

[2.6 Table S6: Keywords used in fulltext screening of all original research papers and meta-analyses 12](#_Toc200538023)

[2.7 Table S7. Quality criteria for evaluation of potentially relevant literature 13](#_Toc200538024)

[2.8 Table S8. Evaluation of relevant publications 20](#_Toc200538025)

[2.9 Table S9. Reported adverse events or undesirable side-effects 31](#_Toc200538026)

[2.10 Table S10. Codes of images used in neurofeedback 38](#_Toc200538027)

[3 Questionnaires 43](#_Toc200538028)

[3.1 Blind check – Patient Version 44](#_Toc200538029)

[3.2 Blind check – Investigator Version 45](#_Toc200538030)

[3.3 Concluding questionnaire 46](#_Toc200538031)

[3.4 SAMmy rating scale 48](#_Toc200538032)

[4 Patient instructions 49](#_Toc200538033)

[4.1 Verbal instructions 49](#_Toc200538034)

[4.2 Written instructions 50](#_Toc200538035)

# Tables

## Table S1. Trial Synopsis

| Full Title | A multi-center, patient-blind and investigator-blind, randomized, parallel-group, superiority study to investigate a neurobiological mechanism of affect instability, comparing four sessions of amygdala fMRI-BOLD neurofeedback with sham feedback in Borderline Personality Disorder  Deutsch: Eine multi-zentrische, patientenverblindete und prüferverblindete, randomisierte Parallelgruppen-Studie zur Erforschung eines neurobiologischen Mechanismus der Affektinstabilität, durch den Vergleich von vier Amygdala-fMRT Neurofeedback Sitzungen mit Sham-Feedback bei der Borderline Persönlichkeitsstörung |
| --- | --- |
| Clinical Investigation Code | Brain Signal Training to Enhance Affect Down-regulation - BrainSTEADy  Deutsch: BrainSTEADy - Hirnsignal-Training zur Verbesserung der Gefühlsregulation |
| Rationale | Individuals with Borderline Personality Disorder (BPD) experience intensive, instable negative emotions. Hyperactivity of the amygdala is assumed to drive exaggerated emotional responses in BPD. Neurofeedback is an endogenous neuromodulation method to address the imbalance of neural circuits. Downregulation of amygdala hyperactivation with neurofeedback may ameliorate dysregulated emotions in BPD. The BrainSTEADy trial is designed to determine whether amygdala-fMRI-BOLD neurofeedback has a specific effect on affect instability in BPD beyond nonspecific benefit. |
| Primary Objective and Endpoint | To determine whether amygdala-fMRI-BOLD neurofeedback has a specific effect on dysregulated affect in BPD beyond nonspecific benefit. The main hypothesis to be tested is whether downregulation training of amygdala activation with neurofeedback reduces the intensity of negative affect assessed before treatment (T0) as compared to after treatment (T1) and whether this change is greater in the treatment group as compared to the control group.  Primary endpoint:  Affect intensity, group difference of change from T0 to T1 measured via experience sampling using ecological momentary assessment (EMA). |
| Main Secondary Objectives and Endpoints | To assess whether downregulation training of amygdala activation with neurofeedback reduces the intensity of negative affect assessed before treatment (T0) as compared to 3-months follow-up (T2). To assess symptom severity and neural regulation success through fMRI-neurofeedback. To investigate the reduction in health economic burden and an improvement in patient reported outcomes in terms of utilities, before treatment (T0) as compared to 6-months follow-up (T3). For all endpoints, we expect that the change is greater in the treatment group as compared to the control group.  Secondary endpoints  A) Affect intensity, group difference of change from T0 to T2.  B) Borderline Symptom Severity, group difference of change from T0 to T1 and …  C) … from T0 to T2, assessed with the Zanarini Rating Scale for BPD, interview version (ZAN-BPD).  D)Amygdala response, group difference of change from T0 to T1.  E) Amygdala self-regulation, group difference of change from T0 to T1  F) Improvement in quality-adjusted life years (QALY), group difference of change from T0 to T3. |
| Clinical Investigation Design | Multi-centre, investigator-blinded, patient-blinded, placebo-controlled, randomised, parallel-group design, prospective study with 2 treatment groups. The individual treatment duration per patient is approximately 20 weeks from screening to 3-months follow-up, with a baseline visit, 4 Neurofeedback visits, and a post-assessment visit. A limited number of scales will be assessed at a second follow-up assessment 6 months after the post-assessment.  This is a two-staged trial with planned interim-analysis after inclusion of 50% of the full sample, and decision to continue recruitment dependent on the interim-analysis result. |
| Sample Size | Stage 1: 82 patients, stage 2: 82 patients |
| Clinical Investigational Population | Inclusion Criteria   1. 18-65 years 2. Diagnosis of Borderline Personality Disorder according to DSM-5 3. Insufficient response to ≥2 therapies. The criterion is fulfilled if the patient reports: 4. 2 or more psychotherapy treatments with 12 or more sessions each OR: 5. 2 or more psychotherapy treatments with a duration per treatment of at least 12 weeks OR: 6. a medical history of 2 or more psychopharmaceutic treatments, each over the course of at least 4 weeks OR: 7. a combination of 2 or more treatments such as:    1. psychotherapy with 12 or more sessions,    2. psychotherapy with a duration of 12 weeks or longer,    3. psychopharmaceutic treatment over the course of at least 4 weeks. 8. Sufficient German language skills to give informed consent to the study, to understand questions posed by used instruments, and capable of completing the fMRI tasks 9. Ability of subject to understand character and individual consequences of clinical investigation 10. Written informed consent (must be available before enrollment in the clinical investigation) 11. For women of childbearing potential (WOCBP) adequate contraception (as defined in Appendix 2).   Exclusion Criteria   1. Treatment with benzodiazepines within 7 days prior the initial screening 2. Current alcohol or substance dependence within 1 month prior the initial screening 3. Meeting the diagnostic criteria for a psychotic disorder or schizophrenia (life-time), as determined by clinical interview at initial screening 4. Current or history of significant neurological condition (such as stroke, traumatic brain injury, space occupying lesions, multiple sclerosis, Parkinson’s disease, vascular dementia, transient ischemic attack) 5. Significant visual impairment that might interfere with the performance of the behavioural tasks or fMRI tasks 6. Change of treatment (psychopharmacologic, psychological) 2 weeks prior to or during the study participation 7. Treatment with any neurofeedback three months prior to or during the study participation. 8. Unable or unwilling to comply with study procedures, including study prohibitions and restrictions 9. History of claustrophobia or inability to tolerate scanner environment 10. Fulfilling any of the MRI contraindications on the standard site radiography screening questionnaire (e.g. history of surgery involving metal implants) 11. Clinically relevant structural brain abnormality as determined by prior MRI scan 12. Planned medical treatment within the study period that might interfere with the study procedures 13. Participants deemed to be at significant risk of serious violence or suicide based on any one of the following:     1. Significant risk of committing violent acts, homicide, or suicide based on history, routine psychiatric status examination, or according to the investigator’s experience OR     2. Any suicide attempt in the past 3 months (i.e., actual attempt, interrupted attempt, aborted attempt) prior to screening and during the screening period OR     3. Any suicidal ideation of type 4 or 5 in the C-SSRS in the past 3 months prior to randomization or during the screening period. 14. BMI of 16.5 or lower 15. Participation in other clinical trials or observation period of competing trials, respectively 16. Previous participation in this trial (Re-Screening possible, c.f. Chapter 4.6.2) 17. Pregnancy and lactation 18. Held in an institution by legal or official order 19. Legally incapacitated. |
| Interventions and Treatments | Experimental intervention: Real-time fMRI neurofeedback from amygdala’s blood oxygenation level dependent (BOLD) signal + negative emotional picture viewing. Instruction to regulate feedback via down-regulation of one’s emotional response. Neurofeedback is a class I device manufactured by BrainInnovation (Maastricht, Netherlands).  Control intervention: Yoked feedback + negative emotional picture viewing. Instruction to regulate feedback via down-regulation of one’s emotional response.  Duration of intervention per patient: Four training sessions within 4 weeks.  Diagnostic instruments: International Personality Disorder Interview (IPDE), Structured Clinical Interview for DSM-5 (SCID-5) |
| Ethical Considerations | Risk-analysis has been conducted according to ISO 14971 and measures mitigating identified risks have been implemented successfully. Risks of neurofeedback using the software Turbo-BrainVoyager MED Borderline Personality Disorder (TBV MED BPD) have been weighted against those from alternative treatments and non-treatment, taking into account benefits from neurofeedback that were identified with literature analysis. Overall, the result of the risk-benefit analysis is positive. Mitigation measures were successfully implemented by the software developer and are reflected in the investigator’s brochure and in this document wherever necessary. Constant monitoring of risks will be implemented during conductance of the trial. Thus, the clinical trial is in line with ethical standards devised by ISO 14155. |
| Number of Sites | 4 |
| Trial duration | Total trial duration: 42 months  Duration of clinical phase: 29 months  Beginning of the preparation phase: Q2 2024  FSI (first subject in) stage 1: Q1 2025  LSI (last subject in) stage 1: Q1 2026  LSO (last subject out) stage 1: Q1 2026  FSI stage 2: Q2 2026  LSI stage 2: Q4 2026  LSO stage 2: Q1 2027  DBL (database lock): Q3 2027  Statistical analyses completed: Q4 2027  Trial report completed: Q4 2027 |
| Financing | Deutsche Forschungsgemeinschaft (PA 3107/4-1, SCHM 1526/26-1) |

## Table S2. Search strategy and search terms with results

| **Used database** | **Final search terms** | **Period of search:  earliest records in the database to 2023-10-10** | | | **Update period of search:  2023-10-01 to 2024-04-30** | | |
| --- | --- | --- | --- | --- | --- | --- | --- |
|  |  | **Filter** | **Date of Search** | **Re- sults** | **Filter** | **Date of Search** | **Re- sults** |
| Pubmed | fmri AND (neurofeedback OR self-regulation OR "self regulation" OR "brain regulation" OR brain-regulation OR "brain control" OR brain-control OR "control brain" OR "activation control" OR activation-control OR "control activation") | Full text,  English | 2023-10-10 | 1185 | Full text, English, publication date: 2023/10/1:2024/4/30 | 2024-04-30 | 61 |
| Web Of Science (Core Collection) | fmri AND neurofeedback  (All Fields) OR fmri AND self-regulation  (All Fields) OR fmri AND "self regulation"  (All Fields) OR fmri AND brain-control  (All Fields) OR fmri AND "brain control"  (All Fields) OR fmri AND "control brain"  (All Fields) OR fmri AND activation-control  (All Fields) OR fmri AND "activation control"  (All Fields) OR fmri AND "control activation"  (All Fields) OR fmri AND brain-regulation  (All Fields) OR fmri AND "brain regulation" | English or German, No Meeting Abstracts | 2023-10-10 | 1220 | English or German, No Meeting Abstracts, Timespan: 2023-10-01 to 2024-04-30 | 2024-04-30 | 49 |
| Embase | ((fmri and neurofeedback) or (fmri and 'self-regulation') or (fmri and 'self regulation') or (fmri and 'brain control') or (fmri and 'brain-control') or (fmri and 'control brain') or (fmri and 'activation control') or (fmri and 'activation-control') or (fmri and 'control activation') or (fmri and 'brain regulation') or (fmri and 'brain-regulation')).af | No Conference Abstratcs | 2023-10-10 | 923 | No Conference Abstratcs, Year: 2023 or 2024, | 2024-04-30 | 111 |
| Cochrane | ((fmri and neurofeedback) or (fmri and 'self-regulation') or (fmri and 'self regulation') or (fmri and 'brain control') or (fmri and 'brain-control') or (fmri and 'control brain') or (fmri and 'activation control') or (fmri and 'activation-control') or (fmri and 'control activation') or (fmri and 'brain regulation') or (fmri and 'brain-regulation')):ti,ab,kw in Trials | Not Pubmed, Not Embase | 2023-10-10 | CT.gov (400)  CINAHL (1) ICTRP (211) | Not Pubmed, Not Embase, publication date between Oct 2023 and Apr 2024 | 2024-04-30 | CT.gov (25) ICTRP (9) |
| ClinicalTrials.gov | Other terms: fmri AND neurofeedback | *see information in section 2.1.2 Abstract Appraisal* | | | Last update posted from 10/01/2023 to 04/30/2024 | 2024-04-30 | 40 |

Table S2: Search strategy and search terms with results

## Table S3. Publications screened on abstract level

Table S3 is provided as Excel Table ‘Supplementary_Table_S3.xlsx’.

## Table S4. Publication type of potentially relevant publications

| **Publication Types of Potentially Relevant Publications** | **Count of Publication Types** |
| --- | --- |
| Original Research Paper | 365 |
| Review | 169 |
| Clinical Trial | 108 |
| Methods Paper | 44 |
| Meta-Analysis | 16 |
| Perspective | 16 |
| Commentary | 11 |
| Design | 9 |
| Opinion | 5 |
| Erratum | 5 |
| Editorial | 4 |
| Theoretical Framework | 4 |
| Data Paper | 1 |
| Book Chapter | 1 |
| **Grand Total** | **758** |

## Table S5. Description of Clinical Population in Potentially Relevant Publications

|  |  | **All Potentially Relevant Publications, N** | **Original Research Publications and Meta-Analyses of Potentially Relevant Publications, N** |
| --- | --- | --- | --- |
| **Clinical Population** | **Healthy** | 241 | 217 |
|  | **Patients (Borderline Personality Disorder)** | 356 (13) | 161 (3) |
|  | **Not relevant or specified** | 161 | 3 |
| **Grand Total** |  | **758** | **381** |

Note: number in (brackets) are items from additional search in April 2024

| **Patient Population** | **All Potentially Relevant Publications, N** | **Original Research Publications and Clinical Trials Considered Potentially Relevant, N** |
| --- | --- | --- |
| **Major Depression (MD)** | 88 | 68 |
| **Posttraumatic Stress Disorder (PTSD)** | 34 | 21 |
| **Schizophrenia** | 21 | 7 |
| **Stroke** | 19 | 12 |
| **Pain** | 19 | 7 |
| **Attention Deficit Hyperactivity Disorder (ADHD)** | 18 | 9 |
| **Alcohol Dependence** | 16 | 7 |
| **Nicotine Dependence** | 15 | 11 |
| **Borderline Personality Disorder (BPD)** | 13 | 3 |
| **Obsessive Compulsive Disorder (OCD)** | 12 | 7 |
| **Addiction** | 11 | 2 |
| **Parkinson's Disease (PD)** | 11 | 5 |
| **Autism Spectrum Disorder** | 10 | 4 |
| **Anxiety Disorder** | 9 | 4 |
| **Obesity** | 8 | 3 |
| **Eating Disorder** | 8 | 2 |
| **Tourette Syndrome (TS)** | 6 | 2 |
| **Affective Disorders** | 6 | 1 |
| **Substance Abuse** | 6 | 1 |
| **Alzheimer** | 5 | 3 |
| **Tinnitus** | 5 | 3 |
| **Traumatic Brain Injury (TBI)** | 3 | 0 |
| **Mild Cognitive Impairment (MCI)** | 3 | 1 |
| **Sleep Disorder** | 2 | 2 |
| **Huntington's Disease** | 3 | 2 |
| **Cocaine Users** | 2 | 1 |
| **Treatment-Resistant Depression** | 2 | 1 |
| **Personality Disorders** | 1 | 0 |
| **Neurodevelopmental Disorders** | 1 | 0 |
| **Phobia** | 1 | 1 |
| **Psychopaths** | 1 | 1 |
| **Bipolar Disorder** | 1 | 0 |
| **Disruptive Behavior Disorder** | 1 | 1 |
| **Epilepsy** | 1 | 1 |
| **Cerebellar Ataxia** | 1 | 0 |

## Table S6: Keywords used in fulltext screening of all original research papers and meta-analyses

| **Adverse events and/or undesirable side effects** | **Dropout information** |
| --- | --- |
| Side effect, Side-effect, negative outcome, Complication, Undesirable event, Unintended consequence, Adverse event, Adverse effect, Injury, Damage, Detriment, Risk, Hazard, Safety, Harm , Harm, Health, Worsening, Declining, Deterioration, Deteriorating, Escalating, Escalation | Dropout, Excluded, drop-out |

## Table S7. Quality criteria for evaluation of potentially relevant literature

This table has been adapted from a set of quality criteria proposed by the Johner Institut GmbH, Germany, in 2023.

| **Integrity of reporting** | **Explanation** |
| --- | --- |
| High integrity of reporting | The study provides a high integrity of reporting. The integrity of reporting permits clear assessment of all relevant aspects, an uncompromised reproducibility of the study based on description of methodology is possible. |
| Acceptable integrity of reporting | The study provides an acceptable integrity of reporting. The integrity of reporting presents sufficient information for a complete critical evaluation, which reflects the usual standard for clinical publications. No relevant information is missing. However, uncompromised reproducibility is not possible. |
| Low integrity of reporting | The study provides a low integrity of reporting. The integrity of reporting presents sufficient information for critical evaluation to some extent. Results need to be weighed carefully, but the reproducibility is not possible. |
| Inadequate | The study provides an inadequate integrity of reporting. The information given for integrity of reporting for critical assessment is not sufficient, relevant aspects are missing. |

| **Type of study / Level of evidence** | **Explanation** |
| --- | --- |
| Systemic review (1a) | The study design is a systematic review/meta analysis. |
| Meta analysis of RCTs (1b) | The study design is a systematic review/meta analysis of randomized controlled study. |
| RCT (2) | The study design is a randomized, controlled trial. |
| Controlled trial (3a) | The study is a controlled clinical study. |
| Prospective study (3b) | The study design is a prospective study with redetermined eligibility criteria and outcome measures. |
| Observational with control (4a) | The study design is an observational study with controls. |
| Retrospective with control (4b) | The study design is a retrospective study with a control group. |
| Case-control with control (4c) | It is a case-control study with control group. |
| Cohort with control (4d) | It is a cohort study with control group. |
| Observational (5a) | The study design is an observational study. |
| Expert opinions (5b) | The study design contains expert opinions. |
| Case series/study (5c) | The study design contains case series/study. |
| Experimental with control (EC) | It is an experimental study with controls. |
| Experimental (E) | It is an experimental study. |

| **Impact Factor** | **Explanation** |
| --- | --- |
| High (JIF > 5) | The paper has a high JIF. |
| Moderate (JIF 1-5) | The paper has a moderate JIF. |
| Low (JIF 0-1) | The paper has a low JIF. |

| **Statistics** | **Explanation** |
| --- | --- |
| Fully appropriate | Statistics are described extensively and can be considered as adequate. |
| Comparably appropriate | Statistics are described transparently and can be considered as adequate. |
| Sufficient (statistic ok, methods not) | The explanation or description of statistical methods is provided but methods are not considered as fully adequate. |
| Sufficient (methods ok, statistic not) | The explanation or description of statistical methods are only partially provided but methods are apparently adequate. |
| Not appropriate | Statistics are not transparently explained. |

| **Bias** | **Explanation** |
| --- | --- |
| Low risk | The study design has a low risk of bias. It includes substantial measures to minimize the potential for bias (study design), the article does not give reason to suspect otherwise. Therefore, results are considered sufficiently reliable. |
| Moderate risk | The study design has a moderate risk of bias. Authors critically discuss interests/arrangements, possible actions are proposed to limit the potential impact of real or apparent conflicts, the publication does not give reason to suspect bias. Therefore, results are most likely reliable. |
| High risk | The study design has a high risk of bias. The study gives reason to suspect a particular bias, study design prone to bias, results are possibly affected to a certain extent. |
| Apparently biased | Conclusions are significantly affected by conflicts of interests. Obvious bias is included. |

| **Reasoning** | **Explanation** |
| --- | --- |
| Plausible/Logic | Authors give plausible/logical reasoning and correct conclusions. |
| Flaws in reasoning | Authors give weaknesses in argument but lack of apparent misinterpretation. |
| Apparent misinterpretation | Authors give major flaws in reasoning and blatant misinterpretation. |

| **Overall assessment** | **Explanation** |
| --- | --- |
| High strength of evidence | The overall assessment of paper quality shows a high strength of evidence. |
| Moderate strength of evidence | The overall assessment of paper quality shows a moderate strength of evidence. |
| Limited strength of evidence | The overall assessment of paper quality shows a limited strength of evidence. |
| Weak strength of evidence | The overall assessment of paper quality shows a weak strength of evidence. |

| **Clinical Safety/Performance** | **Explanation** |
| --- | --- |
| Focus on clinical safety/performance | The statement/conclusion regarding clinical safety and performance is given. |
| Focus partially on clinical safety/performance | The statement/conclusion regarding the clinical safety/performance is limited, but relevant information for the intervention or state of the art is included in the study. |
| Focus not on clinical safety/performance | A statement/conclusion regarding clinical safety/performance is not possible. There is no relevant information included. |

| **Study design** | **Explanation** |
| --- | --- |
| Appropriate | The study design is considered adequate to examine the clinically relevant aspects or to investigate the discussed questions and topics. It is structured and transparent. |
| Comparably appropriate | The study design is considered as adequate, however, study design lacks certain minor requirements or has minor weaknesses regarding implementation. |
| Sufficient | The study design lacks essential major requirements or has major weaknesses regarding implementation. |
| Not appropriate | The study design lacks basic preconditions to examine the clinically relevant aspects or to investigate the discussed questions and has significant weaknesses in implementation. |
| **Clinically relevant effect** | **Explanation** |
| Clinically relevant/High transferability | The effectiveness of the investigated intervention is regarded as a reliable treatment of the primary symptoms. The clinical effect shows a high external validity and transferability, influence on results and perceivable patient outcomes in clinical routine is expected. |
| Not clear | The clinical long-term effect of the investigated intervention is not assessable. External validity and transferability to clinical practice and influence on results and perceivable patient outcomes in clinical routine are not clear. |
| Not clinically relevant | The effect is of no clinical relevance at all. |

| **Confounder** | **Explanation** |
| --- | --- |
| Unlikely | The observed effects can be traced back to the intervention. No obvious confounders were detectable that could have influenced the result. |
| Uncertain | The observed effects can be at least partially traced back to related factors, e.g., other intervention and medication. There are factors detectable that might have an influence on the result but the effect can only be presumed. |
| Likely | Confounders are obvious. Results are considered to have a high probability to be influenced by other factors than the intervention. |

| **Patient population (compared to target population)** | **Explanation** |
| --- | --- |
| High comparability | The patient population is comparable to the target population. |
| Limited comparability | The comparability of the patient population to the target population is limited. |
| Weak comparability | There are major differences between the patient population in this study and the target population which are not justifiable. |
| Not comparable | The comparison of the patient population in the study and the target population is not possible and limitations are not tolerable. |

| **Overall evaluation** | **Explanation** |
| --- | --- |
| Positive | The study confirms the clinical safety and performance of the intervention. |
| Negative | The study does not confirm the clinical safety and performance of the intervention |
| Limited | The conclusion of the study is ambivalent. Statements regarding the clinical safety and performance of the intervention are limited. |

## Table S8. Evaluation of relevant publications

| **Author, Title, Journal, Year** | Paret, C., Kluetsch, R., Zaehringer, J., Ruf, M., Demirakca, T., Bohus, M., Ende, G., & Schmahl, C. (2016). Alterations of amygdala-prefrontal connectivity with real-time fMRI neurofeedback in BPD patients. Social cognitive and affective neuroscience, 11(6), 952–960. <https://doi.org/10.1093/scan/nsw016> |
| --- | --- |
|  |  |
| **Included** | [x] Yes |
|  | [ ] No |
|  |  |
| **If Excluded, Reason** |  |
|  |  |
| **Objective of Study** | To assess the feasibility and the neural effects of four sessions of amygdala-fMRI neurofeedback with Borderline Personality Disorder patients |
|  |  |
| **Design of Study** | Study Design: Pre-Post Study (No Control group) |
| **Number of participants** |  |
|  | [ ] Fully appropriate |
|  | [x] Sufficient |
|  | [ ] Not appropriate |
|  |  |
| **Number (enter number)** | 10 in experimental group, no control group |
| **Male** | 0 in experimental group, no control group |
| **Female** | 10 in experimental group, no control group |
| **Age** | Mean: 33.6, SD: 9.5 |
|  |  |
| **Number of Experimental Sessions** | 4 |
| **Description of Session** | To start each session, a resting-state scan, with instructions to keep one’s eyes open, was acquired (6 mins), followed by an anatomical scan and by the NF training comprising three runs (9 mins per run). After the training, a run was applied without feedback to assess the transfer of learning (transfer run). Each run comprised three experimental conditions (i.e. ‘regulate’, ‘view’ and ‘neutral’), and each condition was presented five times per run in semi-randomized order. The target region was defined anatomically (bilateral amygdala-mask) and functionally refined with dynamic selection of the 30% voxels with highest activation in the 'view' vs. 'neutral' comparison. |
|  |  |
| **Number of Neurofeedback Runs per Session** | 4 |
| **Description of Neurofeedback Run** | In the ‘regulate’ condition, participants were instructed to down-regulate a thermometer displayed at both sides of an aversive picture presented on a computer monitor. In the ‘view’ condition, an aversive picture was also displayed together with feedback, but participants were instructed to respond naturally to the picture content. In the ‘neutral’ condition, scrambled pictures were presented. Feedback was provided via a thermometer display; an orange line in the lower-half of the display screen indicated patients’ baseline amygdala activation during an 8-s rest period, which preceded the picture presentation. |
|  |  |
| **Inclusion Criteria** | BPD according to DSM-IV. Only patients from a 12-weeks residential Dialectical Behavioral Therapy program were eligible to participate. |
|  |  |
| **Exclusion Criteria** | Patients with bipolar disorder, schizophrenia, severe neurological impairment, body weight >120 kg, BMI <16.5 or who had MR incompatibilities were excluded from participation. |
|  |  |
| **Control Intervention** |  |
|  | [x] No Control Condition |
|  | [ ] No Training Control: Treatment as Usual |
|  | [ ] No Training Control: Waiting List |
|  | [ ] Bidirectional-Regulation Control |
|  | [ ] Placebo Control: Feedback from a Related Brain Region |
|  | [ ] Placebo Control: Feedback from an Unrelated Brain Region |
|  | [ ] Placebo Control: Feedback Based on Non-Brain Signals |
|  | [ ] Placebo Control: Yoked Feedback |
|  | [ ] Placebo Control: Artificially Generated Feedback |
|  | [ ] Mental Rehearsal Control: Inside MRI Scanner |
|  | [ ] Mental Rehearsal Control: Outside MRI Scanner |
|  |  |
|  |  |
| **Randomization** | [ ] Yes |
|  | [x] No |
|  | [ ] Not stated |
|  |  |
| **Blinded** | [x] Open-Label |
|  | [ ] Single-Blinded |
|  | [ ] Double-Blinded |
| **Study design** |  |
|  | [x] Appropriate |
|  | [ ] Comparable appropriate |
|  | [ ] Sufficient |
|  | [ ] Not appropriate |
|  |  |
| **Integrity of reporting** |  |
|  | [ ] High integrity of reporting |
|  | [x] Acceptable integrity of reporting |
|  | [ ] Low integrity of reporting |
|  | [ ] Inadequate |
|  |  |
| **Conflict of interest** |  |
|  | [ ] COI statement included: Potential COI reported |
|  | [x] COI statement included: No COI reported |
|  | [ ] COI statement not included: Potential COI |
|  | [ ] COI statement not included: No COI |
|  | [ ] COI statement not included: Undecided |
|  |  |
| **Type of study / Level of evidence** |  |
|  | [ ] Systematic review (1a) |
|  | [ ] Meta analysis of RCTs (1b) |
|  | [ ] RCT (2) |
|  | [ ] Controlled trial (3a) |
|  | [ ] Prospective study (3b) |
|  | [ ] Observational with control (4a) |
|  | [ ] Retrospective with control (4b) |
|  | [ ] Case-control with control (4c) |
|  | [ ] Cohort with control (4d) |
|  | [ ] Observational  (5a) |
|  | [ ] Expert opinion (5b) |
|  | [ ] Case series/study (5c) |
|  | [ ] Experimental with control (EC) |
|  | [x] Experimental (E) |
|  |  |
| **Impact Factor** |  |
|  | [ ] High (JIF > 5) |
|  | [x] Moderate (JIF 1-5) |
|  | [ ] Low (JIF 0-1) |
|  | [ ] Not specified |
|  |  |
| **Statistics** |  |
|  | [ ] Fully appropriate |
|  | [ ] Comparable appropriate |
|  | [x] Sufficient (Statistics ok, Methods not) |
|  | [ ] Sufficient (Methods ok, Statistics not) |
|  | [ ] Not appropriate |
|  |  |
| **Results** | BPD patients down-regulated right amygdala activation but there were no improvements over time. Task-related amygdala-ventromedial prefrontal cortex connectivity was altered across the four sessions, with an increased connectivity when regulating vs viewing pictures. Resting-state amygdala-lateral prefrontal cortex connectivity was altered and dissociation, as well as scores for ‘lack of emotional awareness’, decreased with training. |
| **Dropouts** |  |
|  | [x] Yes |
|  | [ ] No |
|  |  |
| **Dropout Reasons** | Not stated |
| **Undesirable Side-Effects** |  |
|  | [x] Not mentioned |
|  | [ ] No |
|  | [ ] Yes |
|  |  |
| **Description Side-Effects** |  |
| **Bias** |  |
|  | [ ] Low risk |
|  | [x] Moderate risk |
|  | [ ] High risk |
|  | [ ] apparently biased |
|  |  |
| **Reasoning** |  |
|  | [x] Plausible/logic |
|  | [ ] Flaws in reasoning |
|  | [ ] Apparent misinterpretation |
|  |  |
| **Overall assessment** |  |
|  | [ ] High strength of evidence |
|  | [ ] Moderate strength of evidence |
|  | [x] Limited strength of evidence |
|  | [ ] Weak strength of evidence |
|  |  |
| **Follow-Up** | None |
| **Follow-Up Time** |  |
|  | [ ] 1-7 days |
|  | [ ] 8-14 days |
|  | [ ] 14 days -1 month |
|  | [ ] 2 -3 months |
|  | [ ] 4- 6 months |
|  | [ ] > 6 months |
|  |  |
| **Follow-Up Assessment** |  |
|  | [ ] Excellent |
|  | [ ] Good |
|  | [ ] Fair |
|  | [ ] Not sufficient |
|  | [ ] Not performed |
|  | [x] Not applicable |
|  |  |
| **Conclusion** | Patients with BPD could down-regulate amygdala. Feasibility was assessed in a residential psychotherapy setting. Analysis of fMRI data show a modulation of prefrontal-amygdala networks over sessions. Due to the small sample size, the lack of a control group and the limited assessment of performance/safety parameters, conclusions of clinical benefit is not possible. Improvements in outcome measures may be confounded by psychotherapy effects. With regards to current standards of fMRI research the sample size is too small. |
| **Clinically relevant effect** |  |
|  | [ ] Clinically relevant/High transferability |
|  | [x] Not clear |
|  | [ ] Not clinically relevant |
|  |  |
| **Confounder** |  |
|  | [ ] Unlikely |
|  | [ ] Uncertain |
|  | [x] Likely |
|  |  |
| **Device comparison (technical)** |  |
|  | [ ] Device to be evaluated |
|  | [x] Equivalent device |
|  | [ ] Similar device |
|  | [ ] Not named |
|  |  |
| **Device name** | TurboBrainVoyager 3.0 |
|  |  |
| **Medical Device** | [ ] Yes |
|  | [x] No |
| **Device application** |  |
|  | [x] Application comparable |
|  | [ ] Application with limited comparability |
|  | [ ] Application with restricted comparability |
|  | [ ] Application not comparable |
|  |  |
| **Indication** **(compared target indication)** |  |
|  | [x] High comparability |
|  | [ ] Limited comparability |
|  | [ ] Weak comparability |
|  | [ ] Not comparable |
|  |  |
| **Patient population (compared to target population)** |  |
|  | [x] High comparability |
|  | [ ] Limited comparability |
|  | [ ] Weak comparability |
|  | [ ] Not comparable |
|  |  |
| **Clinical Safety/Performance** |  |
|  | [ ] Focus on clinical safety/performance |
|  | [ ] Focus partially on clinical safety/performance |
|  | [x] Focus not on clinical safety/performance |
|  |  |
| **Clinical Performance Evaluation** | Subjects lowered amygdala activation in the 'regulate' vs. 'view' condition (as expected), but no improvement of regulation was seen over time. No significant difference between 'regulate' and 'view' was seen in the transfer runs (i.e., when no feedback was provided for learning assessment). |
| **Clinical Benefit** |  |
|  | [ ] Focus on clinical benefit |
|  | [ ] Focus partially on clinical benefit |
|  | [x] Focus not on clinical benefit |
|  |  |
| **Clinical Benefit Evaluation** | Improvements in dissociation and 'lack of emotional awareness' were seen. Due to the small sample size, statistics of clinical change should be assessed carefully. The aim of this study was not to assess clinical effects, hence definition of primary and secondary endpoints is missing. Further, clinical improvements are likely confounded by residential psychotherapy. |
| **Overall evaluation** |  |
|  | [ ] Positive |
|  | [ ] Negative |
|  | [x] Limited |
|  |  |

| **Author, Title, Journal, Year** | Zaehringer, J., Ende, G., Santangelo, P., Kleindienst, N., Ruf, M., Bertsch, K., Bohus, M., Schmahl, C., & Paret, C. (2019). Improved emotion regulation after neurofeedback: A single-arm trial in patients with borderline personality disorder. NeuroImage. Clinical, 24, 102032. <https://doi.org/10.1016/j.nicl.2019.102032> |
| --- | --- |
|  |  |
| **Included** | [x] Yes |
|  | [ ] No |
|  |  |
| **If Excluded, Reason** |  |
|  |  |
| **Objective of Study** | To estimate effect sizes of change in emotion-regulation-related outcomes achieved through three sessions of real-time fMRI neurofeedback of amygdala activation in Borderline Personality Disorder. |
|  |  |
| **Design of Study** | Pre-Post Study (No Control group) |
| **Number of participants** |  |
|  | [ ] Fully appropriate |
|  | [x] Sufficient |
|  | [ ] Not appropriate |
|  |  |
| **Number (enter number)** | 26 in experimental group, no control group |
| **Male** | 0 in experimental group, no control group |
| **Female** | 26 in experimental group, no control group |
| **Age** | Mean: 33.42 SD: 11.10 |
|  |  |
| **Number of Experimental Sessions** | 3 |
| **Description of Session** | Sessions were administered on 3 different days. Training days were scheduled 2–7 days apart from each other. Each session started with an anatomical scan and the anatomical definition of the right amygdala in subject space. Session 1 started with two functional MRI tasks and was followed by 1 neurofeedback run. In session 2 subjects completed 2 neurofeedback runs. In session 3, following the fourth neurofeedback run, subjects completed again the fMRI tasks. Each session lasted about 45 minutes. |
|  |  |
| **Number of Neurofeedback Runs per Session** | 4 |
| **Description of Neurofeedback Run** | Subjects were instructed to look at negative pictures (without feedback, ‘view’ condition), or downregulate a colored thermometer bar, representing brain activation while watching negative pictures (‘down’ condition), respectively. Participants were not given a particular strategy to downregulate. Rather, they were told to assess what strategy worked best for them. In the ‘view’ condition, a picture with negative emotional content was presented for 18 s, followed by a fixation cross on a grey background (‘rest,’ 12 s). In the ‘down’ condition, pictures were presented with feedback. |
|  |  |
| **Inclusion Criteria** | BPD diagnosis according to DSM-IV, no psychotropic medication or stable psychotropic medication of SSRI/SNRI throughout course of the study |
|  |  |
| **Exclusion Criteria** | Patients were excluded from our study in cases of severe somatic illness and if exclusion criteria related to MRI were fulfilled (metal implants, left-handedness, claustrophobia, and pregnancy). Further exclusion criteria were alcohol or substance abuse within the last 6 months, lifetime psychotic disorder, bipolar affective disorder, or mental retardation. |
|  |  |
| **Control Intervention** |  |
|  | [x] No Control Condition |
|  | [ ] No Training Control: Treatment as Usual |
|  | [ ] No Training Control: Waiting List |
|  | [ ] Bidirectional-Regulation Control |
|  | [ ] Placebo Control: Feedback from a Related Brain Region |
|  | [ ] Placebo Control: Feedback from an Unrelated Brain Region |
|  | [ ] Placebo Control: Feedback Based on Non-Brain Signals |
|  | [ ] Placebo Control: Yoked Feedback |
|  | [ ] Placebo Control: Artifically Generated Feedback |
|  | [ ] Mental Rehearsal Control: Inside MRI Scanner |
|  | [ ] Mental Rehearsal Control: Outside MRI Scanner |
|  |  |
|  |  |
| **Randomization** | [ ] Yes |
|  | [x] No |
|  | [ ] Not stated |
|  |  |
| **Blinded** | [x] Open-Label |
|  | [ ] Single-Blinded |
|  | [ ] Double-Blinded |
| **Study design** |  |
|  | [ ] Appropriate |
|  | [ ] Comparable appropriate |
|  | [x] Sufficient |
|  | [ ] Not appropriate |
|  |  |
| **Integrity of reporting** |  |
|  | [ ] High integrity of reporting |
|  | [x] Acceptable integrity of reporting |
|  | [ ] Low integrity of reporting |
|  | [ ] Inadequate |
|  |  |
| **Conflict of interest** |  |
|  | [ ] COI statement included: Potential COI reported |
|  | [x] COI statement included: No COI reported |
|  | [ ] COI statement not included: Potential COI |
|  | [ ] COI statement not included: No COI |
|  | [ ] COI statement not included: Undecided |
|  |  |
| **Type of study / Level of evidence** |  |
|  | [ ] Systematic review (1a) |
|  | [ ] Meta analysis of RCTs (1b) |
|  | [ ] RCT (2) |
|  | [ ] Controlled trial (3a) |
|  | [ ] Prospective study (3b) |
|  | [ ] Observational with control (4a) |
|  | [ ] Retrospective with control (4b) |
|  | [ ] Case-control with control (4c) |
|  | [ ] Cohort with control (4d) |
|  | [ ] Observational  (5a) |
|  | [ ] Expert opinion (5b) |
|  | [ ] Case series/study (5c) |
|  | [ ] Experimental with control (EC) |
|  | [x] Experimental (E) |
|  |  |
| **Impact Factor** |  |
|  | [ ] High (JIF > 5) |
|  | [x] Moderate (JIF 1-5) |
|  | [ ] Low (JIF 0-1) |
|  | [ ] Not specified |
|  |  |
| **Statistics** |  |
|  | [x] Fully appropriate |
|  | [ ] Comparable appropriate |
|  | [ ] Sufficient (Statistics ok, Methods not) |
|  | [ ] Sufficient (Methods ok, Statistics not) |
|  | [ ] Not appropriate |
|  |  |
| **Results** | Participants were able to downregulate their amygdala blood oxygen-dependent (BOLD) response with neurofeedback. There was a decrease of BPD symptoms as assessed with the Zanarini rating scale for BPD (ZAN-BPD) and a decrease in emotion-modulated startle to negative pictures after training. Further explorative analyses suggest that patients indicated less affective instability, as seen by lower hour-to-hour variability in negative affect and inner tension in daily life. |
| **Dropouts** |  |
|  | [x] Yes |
|  | [ ] No |
|  |  |
| **Dropout Reasons** | Three subjects were allocated to the intervention but discontinued before the full training dose was received. At least one of three subjects had an unexpected hospital stay and therefore terminated participation. For at least one subject two much time passed between the sessions, thus the decision was made to terminate participation. Reporting of reasons of discontinuation is insufficient, detailed information is missing on (1) how many subjects dropped out because of what reason, (2) whether an adverse event was recorded, (3) information about the event and its relation to the psychopathology and to study participation, and (4) whether any adverse event were related to the treatment. |
| **Undesirable Side-Effects** |  |
|  | [x] Not mentioned |
|  | [ ] No |
|  | [ ] Yes |
|  |  |
| **Description Side-Effects** | Three subjects were allocated to the intervention but discontinued before the full training dose was received. At least one of three subjects had an unexpected hospital stay and therefore terminated participation. For at least one subject two much time passed between the sessions, thus the decision was made to terminate participation. Reporting of reasons of discontinuation is insufficient, detailled information is missing on (1) how many subjects dropped out because of what reason, (2) whether an adverse event was recorded, (3) information about the event and its relation to the psychopathology and to study participation, and (4) whether any adverse event were related to the treatment. |
| **Bias** |  |
|  | [ ] Low risk |
|  | [x] Moderate risk |
|  | [ ] High risk |
|  | [ ] apparently biased |
|  |  |
| **Reasoning** |  |
|  | [x] Plausible/logic |
|  | [ ] Flaws in reasoning |
|  | [ ] Apparent misinterpretation |
|  |  |
| **Overall assessment** |  |
|  | [ ] High strength of evidence |
|  | [ ] Moderate strength of evidence |
|  | [x] Limited strength of evidence |
|  | [ ] Weak strength of evidence |
|  |  |
| **Follow-Up** | Clinical, behavioral (questionnaire) and fMRI-outcomes were re-assessed. |
| **Follow-Up Time** |  |
|  | [ ] 1-7 days |
|  | [ ] 8-14 days |
|  | [ ] 14 days -1 month |
|  | [x] 1-2 months |
|  | [ ] 2 -3 months |
|  | [ ] 4- 6 months |
|  | [ ] > 6 months |
|  |  |
| **Follow-Up Assessment** |  |
|  | [x] Excellent |
|  | [ ] Good |
|  | [ ] Fair |
|  | [ ] Not sufficient |
|  | [ ] Not performed |
|  | [ ] Not applicable |
|  |  |
| **Conclusion** | The present study provides the first preliminary empirical basis for informed decision-making in primary outcome measures of larger clinical trials of amygdala neurofeedback training. We show that general BPD psychopathology, as well as different aspects of emotion dysregulation, improve after training, although these effects do not remain statistically significant after a conservative correction for multiple tests. If confirmed by an independent study, our results suggest that the ZAN-BPD, emotion regulation (assessed with emotion-modulated startle), and EMA are appropriate measures to quantify these improvements. |
| **Clinically relevant effect** |  |
|  | [x] Clinically relevant/High transferability |
|  | [ ] Not clear |
|  | [ ] Not clinically relevant |
|  |  |
| **Confounder** |  |
|  | x Unlikely |
|  | [ ] Uncertain |
|  | [ ] Likely |
|  |  |
| **Device comparison (technical)** |  |
|  | [ ] Device to be evaluated |
|  | [ ] Equivalent device |
|  | [x] Similar device |
|  | [ ] Not named |
|  |  |
| **Device name** | in-house programmed with SPM8 toolbox for Matlab |
|  |  |
| **Medical Device** | [ ] Yes |
|  | [x] No |
| **Device application** |  |
|  | [x] Application comparable |
|  | [ ] Application with limited comparability |
|  | [ ] Application with restricted comparability |
|  | [ ] Application not comparable |
|  |  |
| **Indication** **(compared target indication)** |  |
|  | [x] High comparability |
|  | [ ] Limited comparability |
|  | [ ] Weak comparability |
|  | [ ] Not comparable |
|  |  |
| **Patient population (compared to target population)** |  |
|  | [x] High comparability |
|  | [ ] Limited comparability |
|  | [ ] Weak comparability |
|  | [ ] Not comparable |
|  |  |
| **Clinical Safety/Performance** |  |
|  | [ ] Focus on clinical safety/performance |
|  | [x] Focus partially on clinical safety/performance |
|  | [ ] Focus not on clinical safety/performance |
|  |  |
| **Clinical Performance Evaluation** | fMRI-BOLD measures of 'down'-regulation and the 'view' condition (i.e., baseline condition) were compared. Participants regulated amygdala-activation in the 'down' vs. 'view' condition. The effect was significant in the last run (and in one of the analyses also in run 2). The linear effect (i.e., improvement of down-regulation) was not significant. Safety was not evaluated. Transfer of regulation was not assessed. Comparison of pre- vs. post fMRI tasks of spontaneous amygdala-response to emotional stimuli was not significant. Correlations of amygdala-down-regulation and outcomes (behavioral, clinical) was not significant. |
| **Clinical Benefit** |  |
|  | [ ] Focus on clinical benefit |
|  | [x] Focus partially on clinical benefit |
|  | [ ] Focus not on clinical benefit |
|  |  |
| **Clinical Benefit Evaluation** | BPD psychopathology, emotion dysregulation, and affective instability improved at several levels of analysis, including self-report, startle modulation, and experience in everyday life. |
| **Overall evaluation** |  |
|  | [x] Positive |
|  | [ ] Negative |
|  | [ ] Limited |
|  |  |

## Table S9. Reported adverse events or undesirable side-effects

| **Reporting of (absence of) Adverse Events and/or Undesirable Side Effects of NeuroFeedback (NF)** | **Clinical Population** | **Reference** |
| --- | --- | --- |
| “Common side-effects included headache, nausea and drowsiness. These generally did not lead to withdrawal of therapy except in one study.” [p.1440] | Chronic Pain | Patel, K., Sutherland, H., Henshaw, J., Taylor, J. R., Brown, C. A., Casson, A. J., Trujillo-Barreton, N. J., Jones, A. K. P., & Sivan, M. (2020). Effects of neurofeedback in the management of chronic pain: A systematic review and meta-analysis of clinical trials. *European journal of pain (London, England)*, *24*(8), 1440–1457. <https://doi.org/10.1002/ejp.1612> |
| Five of thirtythree studies mentioned side effects. No study reported any adverse effects during NF. Three studies assessed sleepiness, fatigue, or frustration, with no difference in these side effects between the NF and control groups. | Healthy | Onagawa, R., Muraoka, Y., Hagura, N., & Takemi, M. (2023). An investigation of the effectiveness of neurofeedback training on motor performance in healthy adults: A systematic review and meta-analysis. *NeuroImage*, *270*, 120000. <https://doi.org/10.1016/j.neuroimage.2023.120000> |
| An increase in effective connectivity (EC) strength between the supplementary motor area (SMA) and premotor cortex (PMC) was observed, which is generally considered a positive finding for stroke patients. A decreased contralesional premotor and primary motor regions connectivity occurred, which is a common finding which is explained as the “well-known ‘maladaptive’ plasticity mechanism in stroke”, and the authors comment that the influence of the decreased inter-hemispheric connectivity “on motor recovery is debated and insufficiently understood”. [p.9] | Stroke | Giulia, L., Adolfo, V., Julie, C., Quentin, D., Simon, B., Fleury, M., Leveque-Le Bars, E., Bannier, E., Lécuyer, A., Barillot, C., & Bonan, I. (2021). The impact of neurofeedback on effective connectivity networks in chronic stroke patients: an exploratory study. *Journal of neural engineering*, *18*(5), 10.1088/1741-2552/ac291e. <https://doi.org/10.1088/1741-2552/ac291e> |
| After multiple sessions, non-responders to the neurofeedback showed an “aversive effect”: more “socially avoidant behaviour (of happy faces becomes more pronounced with a more exaggerated reduction in reappraisal ability (relative to baseline))”. [p.6] | Healthy | Lisk, S., Kadosh, K. C., Zich, C., Haller, S. P., & Lau, J. Y. (2020). Training negative connectivity patterns between the dorsolateral prefrontal cortex and amygdala through fMRI-based neurofeedback to target adolescent socially-avoidant behaviour. *Behaviour research and therapy*, *135*, 103760. <https://doi.org/10.1016/j.brat.2020.103760> |
| NF happened to coactivate default mode network (DMN) areas, which can be rather harmful in treating patients with major depression (MDD). In other studies an increase of DMN activity has been reported for MDD patients, which was associated with depressive symptoms. | Healthy | Mayeli, A., Misaki, M., Zotev, V., Tsuchiyagaito, A., Al Zoubi, O., Phillips, R., Smith, J., Stewart, J. L., Refai, H., Paulus, M. P., & Bodurka, J. (2020). Self-regulation of ventromedial prefrontal cortex activation using real-time fMRI neurofeedback-Influence of default mode network. *Human brain mapping*, *41*(2), 342–352. <https://doi.org/10.1002/hbm.24805> |
| An increase in anxiety was noticed in some participants of both groups: after NF (n=5) and after sham (n=9). | Healthy | Zhao, Z., Yao, S., Li, K., Sindermann, C., Zhou, F., Zhao, W., Li, J., Lührs, M., Goebel, R., Kendrick, K. M., & Becker, B. (2019). Real-Time Functional Connectivity-Informed Neurofeedback of Amygdala-Frontal Pathways Reduces Anxiety. *Psychotherapy and psychosomatics*, *88*(1), 5–15. <https://doi.org/10.1159/000496057> |
| Increased pain was noticed in some participants after the training, consisting of pain which is unexpected in ipsilateral training (n=4) or pain expected in contralateral training (n=9). | Phantom Limb Pain | Yanagisawa, T., Fukuma, R., Seymour, B., Tanaka, M., Yamashita, O., Hosomi, K., Kishima, H., Kamitani, Y., & Saitoh, Y. (2022). Neurofeedback Training without Explicit Phantom Hand Movements and Hand-Like Visual Feedback to Modulate Pain: A Randomized Crossover Feasibility Trial. *The journal of pain*, *23*(12), 2080–2091. <https://doi.org/10.1016/j.jpain.2022.07.009> |
| Some patients reported a worse mood after NF on the third day (n=2). | Schizophrenia | Dyck, M. S., Mathiak, K. A., Bergert, S., Sarkheil, P., Koush, Y., Alawi, E. M., Zvyagintsev, M., Gaebler, A. J., Shergill, S. S., & Mathiak, K. (2016). Targeting Treatment-Resistant Auditory Verbal Hallucinations in Schizophrenia with fMRI-Based Neurofeedback - Exploring Different Cases of Schizophrenia. *Frontiers in psychiatry*, *7*, 37. <https://doi.org/10.3389/fpsyt.2016.00037> |
| Motor performance decreased in participants with low activity in the contralateral primary motor cortex during neurofeedback-guided motor imagery. | Healthy | Blefari, M. L., Sulzer, J., Hepp-Reymond, M. C., Kollias, S., & Gassert, R. (2015). Improvement in precision grip force control with self-modulation of primary motor cortex during motor imagery. *Frontiers in behavioral neuroscience*, *9*, 18. <https://doi.org/10.3389/fnbeh.2015.00018> |
| Non-lethal overdose of paracetamol (n=1). This event occurred in the `treatment as usual’ group, which was the group not receiving neurofeedback. | Alcohol Dependence | Subramanian, L., Skottnik, L., Cox, W. M., Lührs, M., McNamara, R., Hood, K., Watson, G., Whittaker, J. R., Williams, A. N., Sakhuja, R., Ihssen, N., Goebel, R., Playle, R., & Linden, D. E. J. (2021). Neurofeedback Training versus Treatment-as-Usual for Alcohol Dependence: Results of an Early-Phase Randomized Controlled Trial and Neuroimaging Correlates. *European addiction research*, *27*(5), 381–394. <https://doi.org/10.1159/000513448> |
| Worsening of the ability to control volitional modulation of the activity in the neurofeedback target region; this was explained by the offer of an explicit reward. | Healthy | Direito, B., Ramos, M., Pereira, J., Sayal, A., Sousa, T., & Castelo-Branco, M. (2020). Directly Exploring the Neural Correlates of Feedback-Related Reward Saliency and Valence During Real-Time fMRI-Based Neurofeedback. *Frontiers in human neuroscience*, *14*, 578119. <https://doi.org/10.3389/fnhum.2020.578119> |
| A hand injury occurred after the second localizer session (n=1). From the text, it is unclear how the hand injury occurred and what the cause of the hand injury was. | Healthy | Oblak, E. F., Sulzer, J. S., & Lewis-Peacock, J. A. (2019). A simulation-based approach to improve decoded neurofeedback performance. *NeuroImage*, *195*, 300–310. <https://doi.org/10.1016/j.neuroimage.2019.03.062> |
| “Repeated fMRI scanning and rtfMRI training, consisting of repeated fMRI scanning in conjunction with cognitive strategies and real-time feedback from several regions of interest in multiple brain systems to control brain region activation, were not associated with an increase in adverse event number or severity. These results demonstrate the safety of repetitive fMRI scanning paradigms similar to those in use in many laboratories worldwide, as well as the safety rtfMRI-based training paradigms.” [p.372] | Chronic Pain | Hawkinson, J. E., Ross, A. J., Parthasarathy, S., Scott, D. J., Laramee, E. A., Posecion, L. J., Rekshan, W. R., Sheau, K. E., Njaka, N. D., Bayley, P. J., & deCharms, R. C. (2012). Quantification of adverse events associated with functional MRI scanning and with real-time fMRI-based training. *International journal of behavioral medicine*, *19*(3), 372–381. <https://doi.org/10.1007/s12529-011-9165-6> |
| “Regarding adverse effects of NF, a marginal aggravation in reactive aggression was observed in the NF group, whereas a significant improvement was observed in the TAU group. However, this finding has to be interpreted with caution because the effect was only marginal, limited to one aggression measure, and not compared to an inactive control group.” [p.40] | Disruptive Behavior Disorder | Böttinger, B. W., Aggensteiner, P. M., Hohmann, S., Heintz, S., Ruf, M., Glennon, J., Holz, N. E., Banaschewski, T., Brandeis, D., & Baumeister, S. (2024). Exploring real-time functional magnetic resonance imaging neurofeedback in adolescents with disruptive behavior disorder and callous unemotional traits. *Journal of affective disorders*, *345*, 32–42. <https://doi.org/10.1016/j.jad.2023.10.036> |
| From a total of 17 reports, 1 included fMRI study reported dizziness (2 of 31 participants)) | Patients (ADHD, Epilepsy) | Rahmani E, Rahmanian M, Mansouri K, Mokhayeri Y, Jamalpour Y, et al. Are There any Possible Side Effects of Neurofeedback? A Systematic Literature Review and Meta-analysis. Iran J Psychiatry Behav Sci. 2023;17(3):e138064. <https://doi.org/10.5812/ijpbs-138064> . |
| “Only four of the 17 studies included in this analysis reported on adverse events/complications. In three of these studies, there were no adverse events (..) and one reported on self-injurious behavior in the control group (..).” [p.14-15]  *Remark on dropout*: "The dropout rate of those treated with NF in the systematic review and meta-analysis was 13.2%. This included some NF therapies, which required participants to relive their trauma. A recent meta-analysis on dropout rates of psychological therapies found that those with a trauma focus were significantly associated with a greater dropout rate than those without a trauma focus (18% vs. 14%) (…). Perhaps NF therapy without a trauma focus may help in this regard. Such a NF therapy for PTSD currently exists and is FDA cleared (..)." [p.15] | PTSD | Voigt, J. D., Mosier, M., & Tendler, A. (2024). Systematic review and meta-analysis of neurofeedback and its effect on posttraumatic stress disorder. *Frontiers in psychiatry*, *15*, 1323485. <https://doi.org/10.3389/fpsyt.2024.1323485> |
| "The intraparietal neurofeed­back condition was associated with worsening depression and [...] there was a positive correlation between intra­ parietal neurofeedback success and BDI score change" [p. E238] | Patients (MDD) | Compère, L., Siegle, G. J., Lazzaro, S., Strege, M., Canovali, G., Barb, S., Huppert, T., & Young, K. (2023). Real-time functional magnetic resonance imaging neurofeedback training of amygdala upregulation increases affective flexibility in depression. Journal of psychiatry & neuroscience : JPN, 48(3), E232–E239. <https://doi.org/10.1503/jpn.220208> |
| “Our main finding was the association of negative therapeutic outcome with a broad appearing DMN and a prominent interaction of the DMN with other brain regions including main SN nodes implicating a potential predictive value of functional connectivity in tobacco-dependent patients in the state of early abstinence.” [p. 292] | Patients (Nicotine Dependence) | Paolini, M., Keeser, D., Rauchmann, B. S., Gschwendtner, S., Jeanty, H., Reckenfelderbäumer, A., Yaseen, O., Reidler, P., Rabenstein, A., Engelbregt, H. J., Maywald, M., Blautzik, J., Ertl-Wagner, B., Pogarell, O., Rüther, T., & Karch, S. (2022). Correlations Between the DMN and the Smoking Cessation Outcome of a Real-Time fMRI Neurofeedback Supported Exploratory Therapy Approach: Descriptive Statistics on Tobacco-Dependent Patients. Clinical EEG and neuroscience, 53(4), 287–296. <https://doi.org/10.1177/15500594211062703> |
| “No adverse effects related to tinnitus distress were reported in the fMRI neurofeedback group after intervention. All participants who completed fMRI neurofeedback therapy tolerated the MRI environment. Only two of eight participants from the fMRI neurofeedback group who dropped out of the study (Table S3) reported possible concerns about MRI-related noise.” [p.6] | Patients (Tinnitus) | Gninenko, N., Trznadel, S., Daskalou, D., Gramatica, L., Vanoy, J., Voruz, F., Robyn, C. L., Spadazzi, A., Yulzari, A., Sitaram, R., Van De Ville, D., Senn, P., & Haller, S. (2024). Functional MRI Neurofeedback Outperforms Cognitive Behavioral Therapy for Reducing Tinnitus Distress: A Prospective Randomized Clinical Trial. Radiology, 310(2), e231143. <https://doi.org/10.1148/radiol.231143> |
| The NF was simulated, so software could be removed; as possible side effect was mentioned: "One of the common instructions (a 6-second delay internal model) resulted in anti-learning in our simulation". [p. 17] | Healthy | Oblak, E. F., Lewis-Peacock, J. A., & Sulzer, J. S. (2017). Self-regulation strategy, feedback timing and hemodynamic properties modulate learning in a simulated fMRI neurofeedback environment. PLoS computational biology, 13(7), e1005681. <https://doi.org/10.1371/journal.pcbi.1005681> |

## Table S10. Codes of images used in neurofeedback

| **OriginalCode** | **Valence (1-9)** | **Arousal (1-9)** | **Order** | **Database** |
| --- | --- | --- | --- | --- |
| 251.jpg | 2,54 | 5,75 | 1 | EmoPicS |
| EM0357.jpg | 1,51 | 7,60 | 2 | EmoMadrid |
| 9570.jpg | 1,68 | 6,14 | 3 | IAPS |
| 9635,1.jpg | 1,90 | 6,54 | 4 | IAPS |
| 1033.jpg | 3,87 | 6,13 | 5 | IAPS |
| 9008.jpg | 3,47 | 4,45 | 6 | IAPS |
| A007.bmp | 1,80 | 7,54 | 7 | GAPED |
| 211.jpg | 2,18 | 6,76 | 8 | EmoPicS |
| 223.jpg | 3,11 | 5,43 | 9 | EmoPicS |
| 3215.jpg | 2,51 | 5,44 | 10 | IAPS |
| A072.bmp | 2,98 | 6,03 | 11 | GAPED |
| Animals_033_h.jpg | 2,41 | 6,61 | 12 | NAPS |
| 6230.jpg | 2,37 | 7,35 | 13 | IAPS |
| 2730.jpg | 2,45 | 6,80 | 14 | IAPS |
| 239.jpg | 1,99 | 7,02 | 15 | EmoPicS |
| 253.jpg | 3,96 | 4,90 | 16 | EmoPicS |
| 215.jpg | 2,34 | 6,39 | 17 | EmoPicS |
| 9921.jpg | 2,04 | 6,52 | 18 | IAPS |
| Bloody knife 1.jpg | 2,12 | 5,73 | 19 | OASIS |
| 8485.jpg | 2,73 | 6,46 | 20 | IAPS |
| A123.bmp | 2,80 | 6,01 | 21 | GAPED |
| 9920.jpg | 2,50 | 5,76 | 22 | IAPS |
| 9419.jpg | 2,55 | 5,19 | 23 | IAPS |
| 6570,1.jpg | 2,54 | 6,12 | 24 | IAPS |
| 9423.jpg | 2,61 | 5,66 | 25 | IAPS |
| 235.jpg | 2,17 | 6,61 | 26 | EmoPicS |
| 219.jpg | 2,98 | 5,73 | 27 | EmoPicS |
| EM0484.jpg | 1,93 | 7,72 | 28 | EmoMadrid |
| 3180.jpg | 1,92 | 5,77 | 29 | IAPS |
| 9342.jpg | 2,85 | 4,49 | 30 | IAPS |
| 6311.jpg | 2,58 | 4,95 | 31 | IAPS |
| 9185.jpg | 1,97 | 5,65 | 32 | IAPS |
| 9620.jpg | 2,70 | 6,11 | 33 | IAPS |
| 3250.jpg | 3,78 | 6,29 | 34 | IAPS |
| EM0394.jpg | 2,99 | 7,71 | 35 | EmoMadrid |
| 2691.jpg | 3,04 | 5,85 | 36 | IAPS |
| EM0090.jpg | 2,22 | 7,66 | 37 | EmoMadrid |
| 208.jpg | 2,67 | 5,82 | 38 | EmoPicS |
| 9250.jpg | 2,57 | 6,60 | 39 | IAPS |
| Doctor 7.jpg | 4,12 | 5,55 | 40 | OASIS |
| 9075.jpg | 1,66 | 6,04 | 41 | IAPS |
| 6940.jpg | 3,53 | 5,35 | 42 | IAPS |
| 9905.jpg | 2,55 | 5,93 | 43 | IAPS |
| Animals_074_h.jpg | 1,70 | 7,37 | 44 | NAPS |
| 229.jpg | 2,81 | 6,72 | 45 | EmoPicS |
| 213.jpg | 2,30 | 6,77 | 46 | EmoPicS |
| 9414.jpg | 2,06 | 6,49 | 47 | IAPS |
| Car crash 3.jpg | 2,37 | 5,88 | 48 | OASIS |
| 248.jpg | 1,67 | 7,27 | 49 | EmoPicS |
| 9429.jpg | 2,68 | 5,63 | 50 | IAPS |
| 9181.jpg | 2,26 | 5,39 | 51 | IAPS |
| Dog attack 3.jpg | 3,20 | 6,45 | 52 | OASIS |
| 214.jpg | 2,24 | 6,55 | 53 | EmoPicS |
| A073.bmp | 1,90 | 6,14 | 54 | GAPED |
| A099.bmp | 1,83 | 6,48 | 55 | GAPED |
| EM0286.jpg | 2,05 | 8,06 | 56 | EmoMadrid |
| A001.bmp | 1,21 | 7,06 | 57 | GAPED |
| 227.jpg | 2,46 | 6,67 | 58 | EmoPicS |
| 3016.jpg | 1,90 | 5,82 | 59 | IAPS |
| 2683.jpg | 2,62 | 6,21 | 60 | IAPS |
| 217.jpg | 3,59 | 5,93 | 61 | EmoPicS |
| 221.jpg | 4,21 | 4,74 | 62 | EmoPicS |
| 2981.jpg | 2,76 | 5,97 | 63 | IAPS |
| EM0122.jpg | 1,66 | 7,18 | 64 | EmoMadrid |
| Angry face 3.jpg | 3,00 | 4,55 | 65 | OASIS |
| 9042.jpg | 3,15 | 5,78 | 66 | IAPS |
| 224.jpg | 2,92 | 4,94 | 67 | EmoPicS |
| 9910.jpg | 2,06 | 6,20 | 68 | IAPS |
| 2753.jpg | 3,17 | 4,29 | 69 | IAPS |
| 9901.jpg | 2,27 | 5,70 | 70 | IAPS |
| 2053.jpg | 2,47 | 5,25 | 71 | IAPS |
| 9433.jpg | 1,84 | 5,89 | 72 | IAPS |
| Sad face 9.jpg | 2,75 | 5,35 | 73 | OASIS |
| 9428.jpg | 2,31 | 5,66 | 74 | IAPS |
| Dead bodies 1.jpg | 1,26 | 5,69 | 75 | OASIS |
| 1930.jpg | 3,79 | 6,42 | 76 | IAPS |
| 222.jpg | 2,89 | 5,58 | 77 | EmoPicS |
| 245.jpg | 2,57 | 6,08 | 78 | EmoPicS |
| 228.jpg | 2,82 | 5,78 | 79 | EmoPicS |
| 230.jpg | 2,86 | 6,36 | 80 | EmoPicS |
| 9530.jpg | 2,93 | 5,20 | 81 | IAPS |
| 6563.jpg | 1,77 | 6,85 | 82 | IAPS |
| 220.jpg | 2,74 | 5,98 | 83 | EmoPicS |
| A008.bmp | 2,13 | 5,94 | 84 | GAPED |
| 242.jpg | 1,40 | 7,99 | 85 | EmoPicS |
| 9600.jpg | 2,48 | 6,46 | 86 | IAPS |
| 2694.jpg | 3,55 | 5,05 | 87 | IAPS |
| 243.jpg | 1,92 | 7,07 | 88 | EmoPicS |
| 9561.jpg | 2,68 | 4,79 | 89 | IAPS |
| 3300.jpg | 2,74 | 4,55 | 90 | IAPS |
| 3230.jpg | 2,02 | 5,41 | 91 | IAPS |
| 9325.jpg | 1,89 | 6,01 | 92 | IAPS |
| 9290.jpg | 2,88 | 4,40 | 93 | IAPS |
| 2795.jpg | 3,92 | 4,70 | 94 | IAPS |
| 6220.jpg | 3,10 | 5,89 | 95 | IAPS |
| 3185.jpg | 2,81 | 5,48 | 96 | IAPS |
| 6561.jpg | 3,16 | 4,99 | 97 | IAPS |
| EM0313.jpg | 1,38 | 8,39 | 98 | EmoMadrid |
| 244.jpg | 2,21 | 7,07 | 99 | EmoPicS |
| 9321.jpg | 2,81 | 6,24 | 100 | IAPS |
| 6562.jpg | 3,19 | 5,08 | 101 | IAPS |
| 1201.jpg | 3,55 | 6,36 | 102 | IAPS |
| 9405.jpg | 1,83 | 6,08 | 103 | IAPS |
| 234.jpg | 1,74 | 6,88 | 104 | EmoPicS |
| 9400.jpg | 2,50 | 5,99 | 105 | IAPS |
| 3220.jpg | 2,49 | 5,52 | 106 | IAPS |
| EM0326.jpg | 1,82 | 8,01 | 107 | EmoMadrid |
| 2095.jpg | 1,75 | 5,25 | 108 | IAPS |
| 3530.jpg | 1,80 | 6,82 | 109 | IAPS |
| 225.jpg | 2,74 | 5,41 | 110 | EmoPicS |
| 9163.jpg | 2,10 | 6,53 | 111 | IAPS |
| 2458.jpg | 4,69 | 5,28 | 112 | IAPS |
| Baby 7.jpg | 3,63 | 5,33 | 113 | OASIS |
| 2457.jpg | 3,20 | 4,94 | 114 | IAPS |
| 3103.jpg | 2,07 | 6,06 | 115 | IAPS |
| 9413.jpg | 1,76 | 6,81 | 116 | IAPS |
| Dog 31.jpg | 2,44 | 5,27 | 117 | OASIS |
| 6312.jpg | 2,48 | 6,37 | 118 | IAPS |
| 3010.jpg | 1,71 | 7,16 | 119 | IAPS |
| 210.jpg | 2,59 | 6,21 | 120 | EmoPicS |
| EM0376.jpg | 2,71 | 7,27 | 121 | EmoMadrid |
| 9300.jpg | 2,26 | 6,00 | 122 | IAPS |
| 9040.jpg | 1,67 | 5,82 | 123 | IAPS |
| 6520.jpg | 1,94 | 6,59 | 124 | IAPS |
| 9230.jpg | 3,89 | 5,77 | 125 | IAPS |
| 9254.jpg | 2,03 | 6,04 | 126 | IAPS |
| 9432.jpg | 2,56 | 4,92 | 127 | IAPS |
| 250.jpg | 2,30 | 6,53 | 128 | EmoPicS |
| 233.jpg | 1,77 | 7,32 | 129 | EmoPicS |
| 212.jpg | 2,48 | 6,13 | 130 | EmoPicS |
| EM0418.jpg | 2,46 | 7,07 | 131 | EmoMadrid |
| 216.jpg | 2,68 | 6,47 | 132 | EmoPicS |
| 237.jpg | 1,57 | 7,72 | 133 | EmoPicS |
| 9410.jpg | 1,51 | 7,07 | 134 | IAPS |
| 9332.jpg | 2,25 | 5,34 | 135 | IAPS |
| 2456.jpg | 2,84 | 4,55 | 136 | IAPS |
| Dummy 1.jpg | 1,21 | 6,60 | 137 | OASIS |
| 9006.jpg | 2,34 | 5,76 | 138 | IAPS |
| 9902.jpg | 2,33 | 6,00 | 139 | IAPS |
| EM0280.jpg | 2,52 | 7,35 | 140 | EmoMadrid |
| Animals_008_v.jpg | 2,63 | 6,80 | 141 | NAPS |
| EM0331.jpg | 1,52 | 7,88 | 142 | EmoMadrid |
| 2718.jpg | 3,65 | 4,46 | 143 | IAPS |
| EM0447.jpg | 2,12 | 7,94 | 144 | EmoMadrid |
| 9926.jpg | 3,85 | 4,83 | 145 | IAPS |
| 1300.jpg | 3,55 | 6,79 | 146 | IAPS |
| 252.jpg | 2,21 | 6,12 | 147 | EmoPicS |
| Dog 26.jpg | 1,40 | 6,14 | 148 | OASIS |
| 9421.jpg | 2,21 | 5,04 | 149 | IAPS |
| 9584.jpg | 3,34 | 4,92 | 150 | IAPS |
| 3350.jpg | 1,88 | 5,72 | 151 | IAPS |
| 3550.jpg | 2,54 | 5,92 | 152 | IAPS |
| 1120.jpg | 3,79 | 6,93 | 153 | IAPS |
| 3195.jpg | 2,06 | 6,36 | 154 | IAPS |
| 232.jpg | 2,11 | 6,42 | 155 | EmoPicS |
| 9415.jpg | 2,82 | 4,91 | 156 | IAPS |
| 9050.jpg | 2,43 | 6,36 | 157 | IAPS |
| 2900.jpg | 2,45 | 5,09 | 158 | IAPS |
| 3500.jpg | 2,21 | 6,99 | 159 | IAPS |
| 3030.jpg | 1,91 | 6,76 | 160 | IAPS |
| 9810.jpg | 2,09 | 6,62 | 161 | IAPS |
| 9435.jpg | 2,27 | 5,00 | 162 | IAPS |
| Animals_052_v.jpg | 4,04 | 6,80 | 163 | NAPS |
| 241.jpg | 1,85 | 6,98 | 164 | EmoPicS |
| 6834.jpg | 2,91 | 6,28 | 165 | IAPS |
| 254.jpg | 2,64 | 5,74 | 166 | EmoPicS |
| 226.jpg | 2,81 | 5,50 | 167 | EmoPicS |
| 9424.jpg | 2,87 | 5,78 | 168 | IAPS |
| Animals_001_h.jpg | 2,57 | 6,44 | 169 | NAPS |
| 6313.jpg | 1,98 | 6,94 | 170 | IAPS |
| A116.bmp | 1,88 | 6,89 | 171 | GAPED |
| A041.bmp | 1,11 | 7,71 | 172 | GAPED |
| 2800.jpg | 1,78 | 5,49 | 173 | IAPS |
| 6510.jpg | 2,46 | 6,96 | 174 | IAPS |
| 9253.jpg | 2,00 | 5,53 | 175 | IAPS |
| 9480.jpg | 3,51 | 5,57 | 176 | IAPS |
| 2799.jpg | 2,42 | 5,02 | 177 | IAPS |
| 6231.jpg | 2,49 | 6,82 | 178 | IAPS |
| 249.jpg | 2,46 | 6,36 | 179 | EmoPicS |
| 8480.jpg | 3,70 | 6,28 | 180 | IAPS |
| 6022.jpg | 2,14 | 6,09 | 181 | IAPS |
| EM0396.jpg | 2,37 | 7,96 | 182 | EmoMadrid |
| 9830.jpg | 2,54 | 4,86 | 183 | IAPS |
| 9800.jpg | 2,04 | 6,05 | 184 | IAPS |
| Animals_037_h.jpg | 3,03 | 6,69 | 185 | NAPS |
| EM0070.jpg | 2,54 | 6,80 | 186 | EmoMadrid |
| 9520.jpg | 2,46 | 5,41 | 187 | IAPS |
| A075.bmp | 1,03 | 8,20 | 188 | GAPED |
| 2301.jpg | 2,78 | 4,57 | 189 | IAPS |
| 9471.jpg | 3,16 | 4,48 | 190 | IAPS |
| 8230.jpg | 2,95 | 5,91 | 191 | IAPS |
| 247.jpg | 2,30 | 6,10 | 192 | EmoPicS |
| 6831.jpg | 2,59 | 5,55 | 193 | IAPS |
| 325.jpg | 3,07 | 6,63 | 194 | EmoPicS |
| 6350.jpg | 1,90 | 7,29 | 195 | IAPS |
| 9412.jpg | 1,83 | 6,72 | 196 | IAPS |
| Animals_053_h.jpg | 3,49 | 5,73 | 197 | NAPS |
| EM0420.jpg | 2,85 | 6,21 | 198 | EmoMadrid |
| A120.bmp | 1,97 | 6,77 | 199 | GAPED |
| 3181.jpg | 2,30 | 5,06 | 200 | IAPS |
| Animals_016_h.jpg | 2,67 | 6,92 | 201 | NAPS |
| Animal carcass 6.jpg | 2,86 | 5,96 | 202 | OASIS |
| 218.jpg | 2,86 | 5,52 | 203 | EmoPicS |
| 2700.jpg | 3,19 | 4,77 | 204 | IAPS |
| 231.jpg | 2,19 | 6,56 | 205 | EmoPicS |
| 1202.jpg | 3,35 | 5,94 | 206 | IAPS |
| 6415.jpg | 2,21 | 6,20 | 207 | IAPS |
| 3160.jpg | 2,63 | 5,35 | 208 | IAPS |
| 9426.jpg | 3,08 | 5,28 | 209 | IAPS |
| 9252.jpg | 1,98 | 6,64 | 210 | IAPS |
| 6370.jpg | 2,70 | 6,44 | 211 | IAPS |
| 6021.jpg | 2,21 | 6,06 | 212 | IAPS |
| 2375,1.jpg | 2,20 | 4,88 | 213 | IAPS |
| 2345,1.jpg | 2,26 | 5,50 | 214 | IAPS |
| 2703.jpg | 1,91 | 5,78 | 215 | IAPS |
| Destruction 2.jpg | 2,58 | 3,75 | 216 | OASIS |
| A095.bmp | 1,51 | 7,06 | 217 | GAPED |
| Dog 24.jpg | 2,19 | 6,03 | 218 | OASIS |
| EM0319.jpg | 1,96 | 7,70 | 219 | EmoMadrid |
| 9425.jpg | 2,67 | 5,92 | 220 | IAPS |
| 246.jpg | 2,90 | 5,65 | 221 | EmoPicS |
| 9183.jpg | 1,69 | 6,58 | 222 | IAPS |
| 3212.jpg | 2,79 | 6,57 | 223 | IAPS |
| 2205.jpg | 1,95 | 4,53 | 224 | IAPS |

# Questionnaires

In this section we provide questionnaires that are not available elsewhere in the literature, or that have not been published in German language before the date of publication of this article.

| **Anleitung:**  In dieser Studie werden zwei Versuchsgruppen verglichen. Eine Gruppe erhält direktes Amygdala-Neurofeedback, die Vergleichsgruppe nicht. **Welcher Gruppe, glauben Sie, wurden Sie zugeordnet? Wenn Sie sich nicht sicher sind, dann versuchen Sie, zu raten.**  Bitte beachten Sie: Wir können Ihnen die Gruppenzuordnung zum jetzigen Zeitpunkt noch nicht verraten. Eine vollumfängliche Aufklärung ist erst nach Abschluss der letzten Erhebung (Follow up 2) möglich. | |
| --- | --- |
| Q1: Ich bin in der ….  (Machen Sie eine Angabe) | Amygdala-Neurofeedback Gruppe  Vergleichsgruppe |
| Q2: Wie sicher sind Sie, dass Sie richtigliegen?  (Machen Sie eine Angabe) | überhaupt nicht  eher nicht  eher sicher  sehr sicher |
| Q3: Warum sind Sie sicher/unsicher, dass Sie in dieser Gruppe sind?  Bitte beschreiben Sie. |  |
| Q4: Wenn Sie mögen, dann können Sie hier einen kurzen Text schreiben und Ihre Einschätzung erklären |  |

## Blind check – Patient Version

## Blind check – Investigator Version

| **Bitte geben Sie eine Einschätzung ab, welcher Versuchsgruppe die PatientIn zugeordnet wurde.**  Fragen Sie NICHT die PatientIn nach ihrer/seiner Einschätzung. Besprechen Sie diese Frage auch nicht mit der PatientIn. Wenn Ihre PatientIn Sie auf die Gruppenzuordnung anspricht, dann gehen Sie bitte im Gespräch nicht näher darauf ein. Sie dürfen der PatientIn mitteilen, dass Sie die Zuordnung selbst nicht kennen und angewiesen sind, zum jetzigen Zeitpunkt nicht mit der PatientIn darüber zu sprechen. | |
| --- | --- |
| Q1: PatientIn ist in der …  (Machen Sie eine Angabe) | Amygdala-Neurofeedback Gruppe  Vergleichsgruppe |
| Q2: Wie sicher sind Sie, dass Sie richtigliegen?  (Machen Sie eine Angabe) | überhaupt nicht  eher nicht  eher sicher  sehr sicher |
| Q3: Warum sind Sie sicher/unsicher, dass die PatientIn in dieser Gruppe ist?  Bitte beschreiben Sie. |  |
| Q4: Einschätzung basiert auf Angaben des/der…  (Mehrfachauswahl) | PrüferIn  NF-TrainerIn  PatientIn  andere Person, nämlich: _______________________ |

## Concluding questionnaire

**German: Neurofeedback Abschluss-Fragebogen**

Wir möchten gern erfahren, wie Sie das Neurofeedback-Training erlebt haben. Kreuzen Sie pro Aussage nur eine Antwort an.

1 = Ich stimme überhaupt nicht zu

2 = Ich stimme nicht zu

3 = Ich stimme eher nicht zu

4 = Weder noch / neutral

5 = Ich stimme eher zu

6 = Ich stimme zu

7 = Ich stimme völlig zu

|  | 1 | 2 | 3 | 4 | 5 | 6 | 7 |
| --- | --- | --- | --- | --- | --- | --- | --- |
| Ich habe durch das Neurofeedback gelernt, meine Gefühle zu regulieren. |  |  |  |  |  |  |  |
| Es geht mir besser, verglichen damit, wie es vor der Studienteilnahme war. |  |  |  |  |  |  |  |
| Ich empfand das Neurofeedback-Training als hilfreich. |  |  |  |  |  |  |  |
| Die Neurofeedback-Sitzungen dauern zu lang. |  |  |  |  |  |  |  |
| Die Bilder beim Neurofeedback-Training waren belastend. |  |  |  |  |  |  |  |
| Die MRT-Messungen waren belastend. |  |  |  |  |  |  |  |
| Das Neurofeedback-Training hat die Symptome meiner Borderline-Störung gebessert. |  |  |  |  |  |  |  |
| Ich habe das Neurofeedback-Training gerne gemacht. |  |  |  |  |  |  |  |
| Ich konnte im Alltag umsetzen, was ich im Neurofeedback gelernt habe. |  |  |  |  |  |  |  |

Wenn Sie wollen, können Sie Ihre Angaben unten näher erklären. Teilen Sie uns Ihre eigenen Erfahrungen als StudienteilnehmerIn mit. Geben Sie uns eine Rückmeldung zum Neurofeedback-Training oder zur Studie.

| *Freitext* |
| --- |

## SAMmy rating scale

The SAMmy rating scale is an in-house adapted version of the SAM scale. For assessment of arousal in context of Borderline Personality Disorder (BPD) we liberally translated the English term ‘arousal’ with the German word ‘Anspannung’, which actually re-translates to ‘tension’. ‘Aversive inner tension’ (German: unangenehme innere Anspannung) is a clinically significant concept in BPD psychopathology, and seems more appropriate than e.g. the German word ‘Erregung’, which, other than the English ‘arousal’, has a sexual connotation.


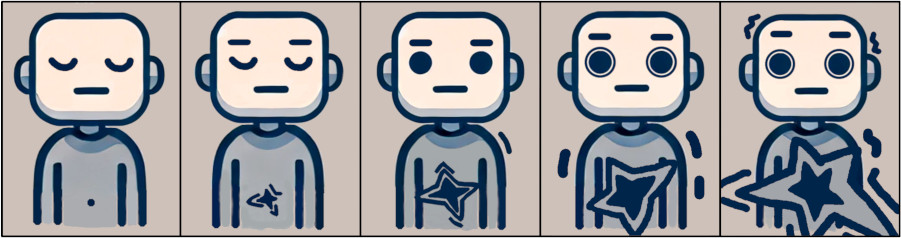


Keine Anspannung sehr hohe Anspannung

# Patient instructions

## Verbal instructions

These instructions will be read out loud to the patient in the beginning of the session:

English version (translation):

During the neurofeedback training, you will try out various mental strategies to reduce the activity in your amygdala. The amygdala is responsible for the regulation of fear and negative emotions. Your aim will be to find out which strategies work best for you to reduce amygdala activity.

A mental strategy is a specific way of thinking or focussing attention to achieve a desired mental or emotional outcome. It involves consciously directing thoughts, feelings or mental processes to influence brain activity. These strategies can target different areas of the brain and their effectiveness can vary depending on the individual and context.

Please make sure you understand the difference between mental and behavioural strategies. For example, you may not actively change your breathing, which would be a behavioural strategy. Instead, you can simply observe your breathing carefully without influencing it. That would be a mental strategy.

German version:

Während des Neurofeedback-Trainings werden Sie verschiedene mentale Strategien ausprobieren, um die Aktivität in Ihrer Amygdala zu reduzieren. Die Amygdala ist für die Regulation von Angst und negativen Emotionen zuständig. Ihr Ziel wird es sein, herauszufinden, welche Strategien für Sie am besten zur Reduktion der Amygdala-Aktivität führen.

Eine mentale Strategie ist eine gezielte Denkweise oder Fokussierung der Aufmerksamkeit, um ein gewünschtes mentales oder emotionales Ergebnis zu erzielen. Sie umfasst das bewusste Lenken von Gedanken, Gefühlen oder mentalen Prozessen, um die Gehirnaktivität zu beeinflussen. Diese Strategien können verschiedene Gehirnregionen ansprechen, und ihre Wirksamkeit kann je nach Individuum und Kontext variieren.

Achten Sie bitte darauf, den Unterschied zwischen mentaler und Verhaltensstrategie zu verstehen. Zum Beispiel dürfen Sie Ihre Atmung nicht aktiv verändern, das wäre eine Verhaltensstrategie. Stattdessen können Sie Ihre Atmung einfach nur aufmerksam beobachten, ohne sie zu beeinflussen. Das wäre eine mentale Strategie.

## Written instructions

The following slides will played to the patient following verbal instructions while the patient is still outside the MR scanner. They include an illustrative movie of a (simplified) neurofeedback cycle and interactive ratings. Original slides with German instructions are provided only.


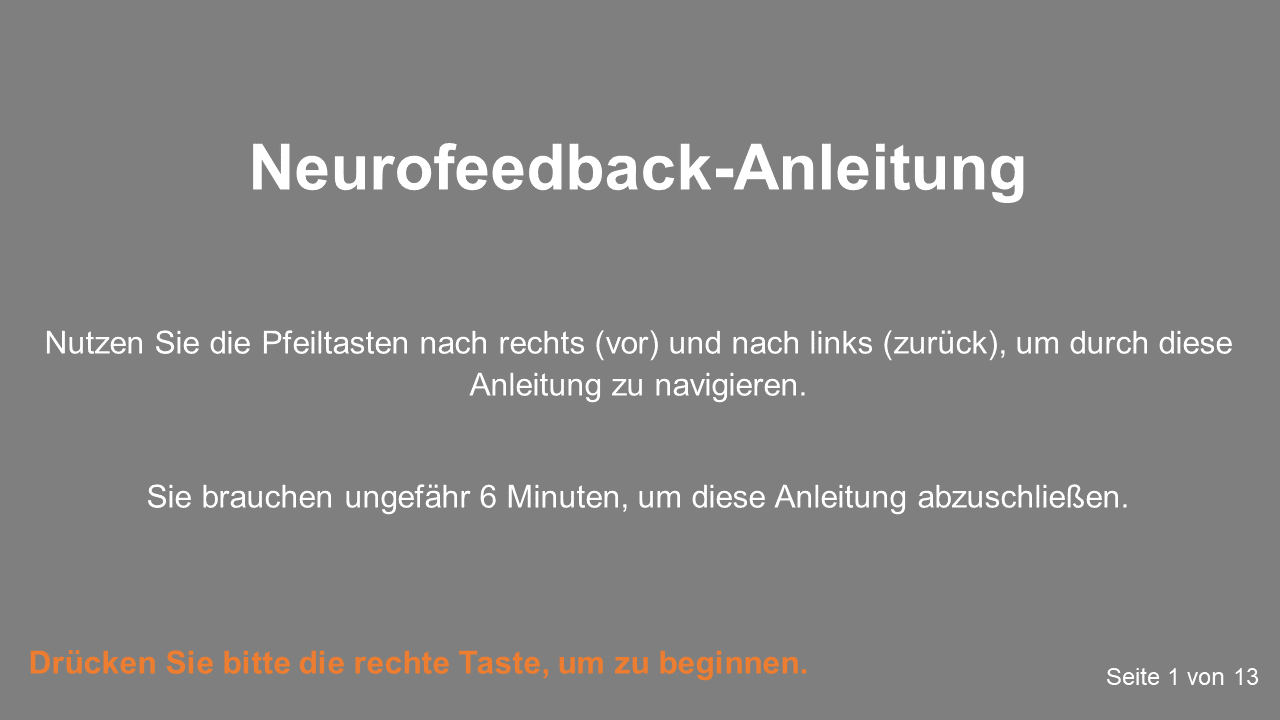


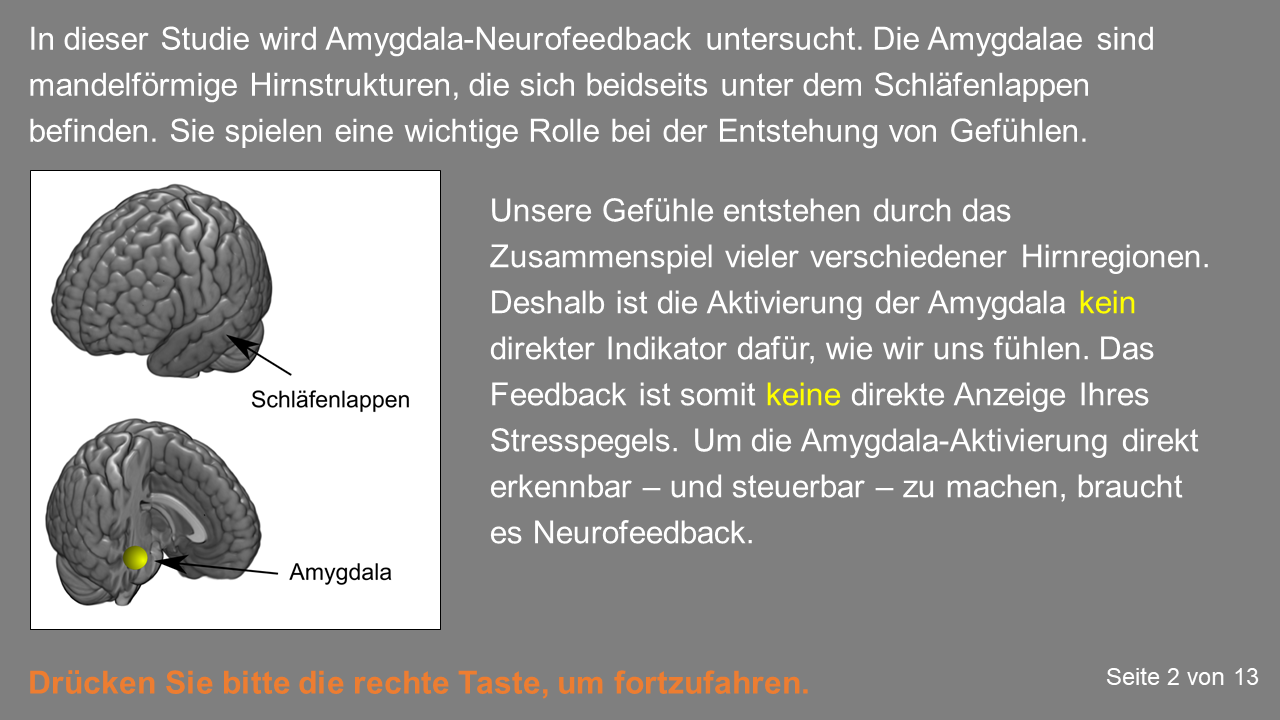

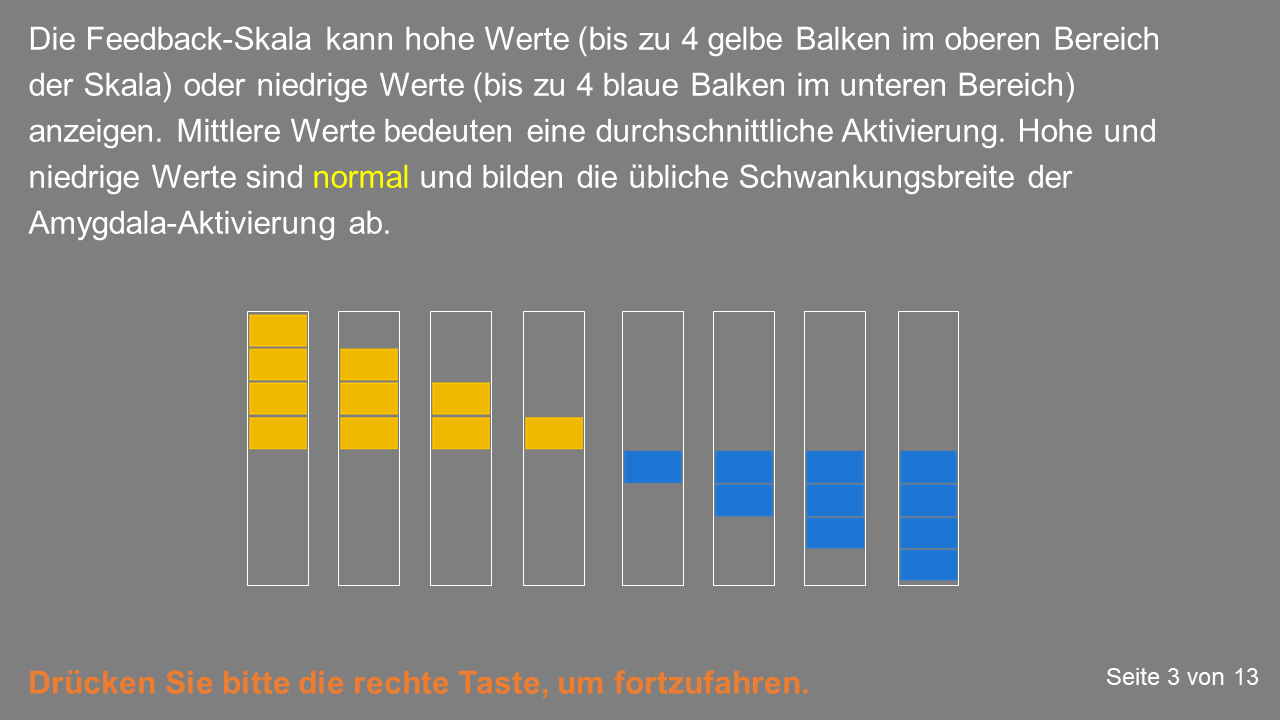


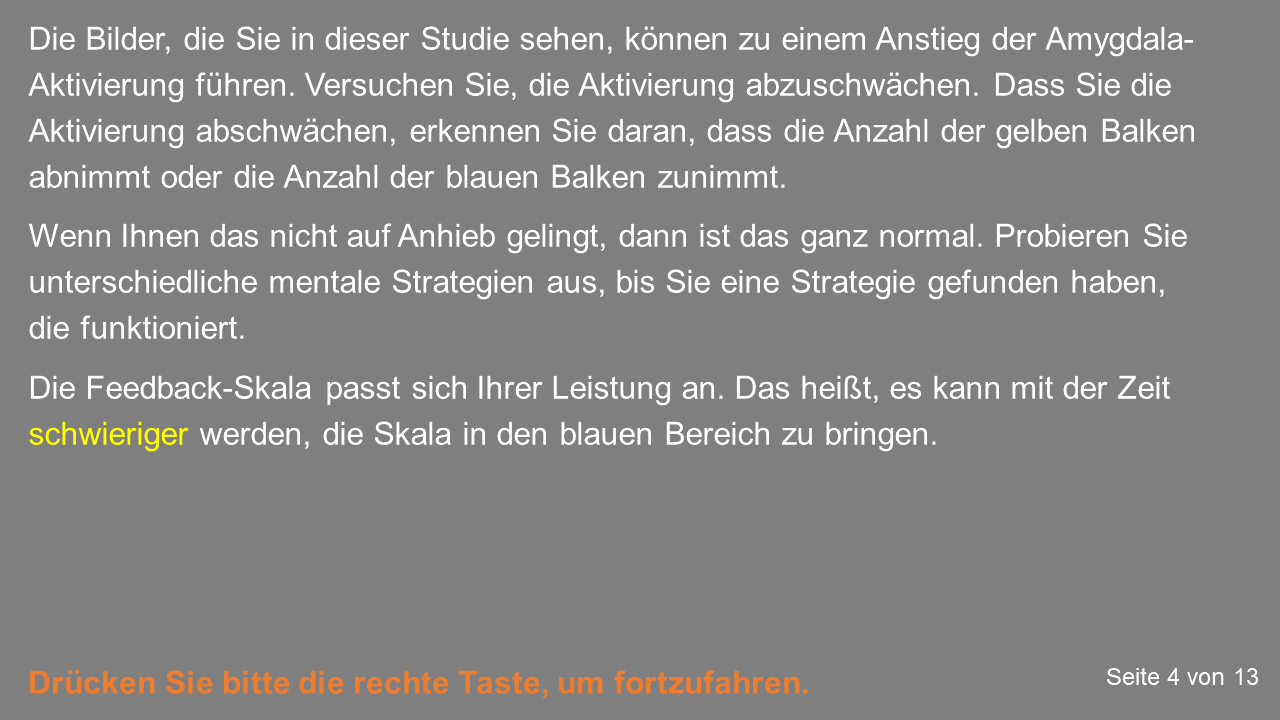


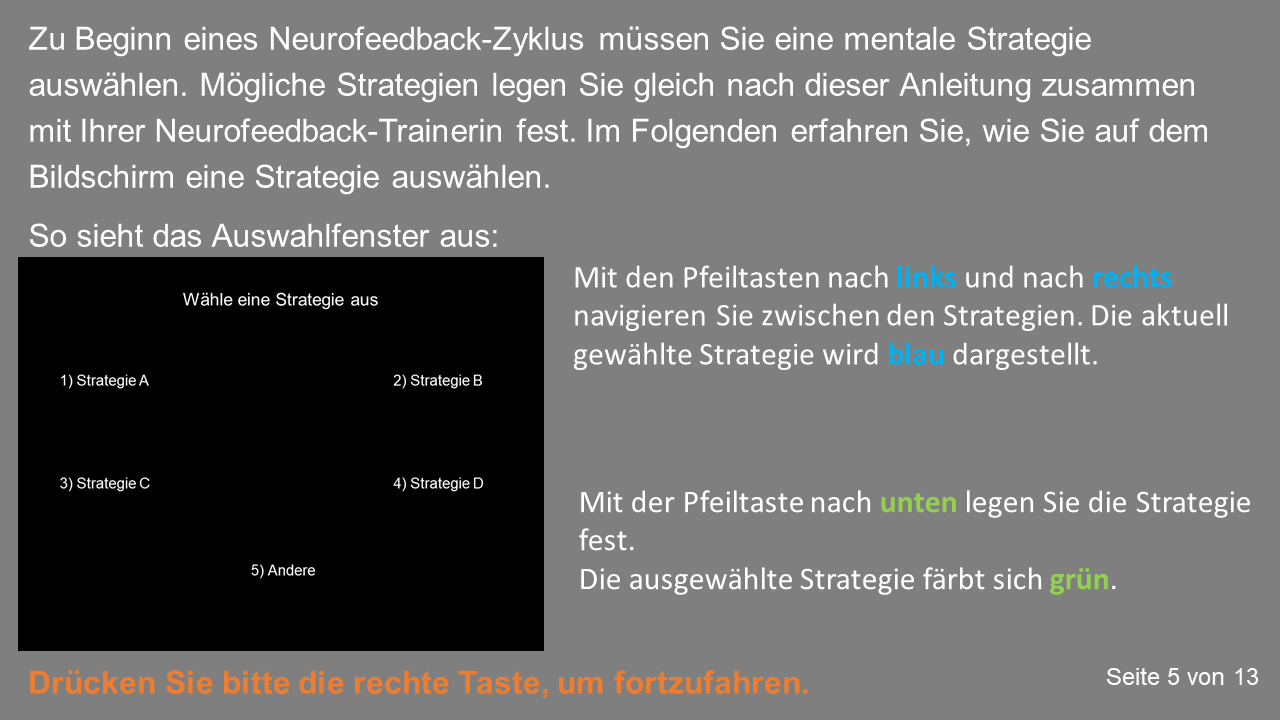


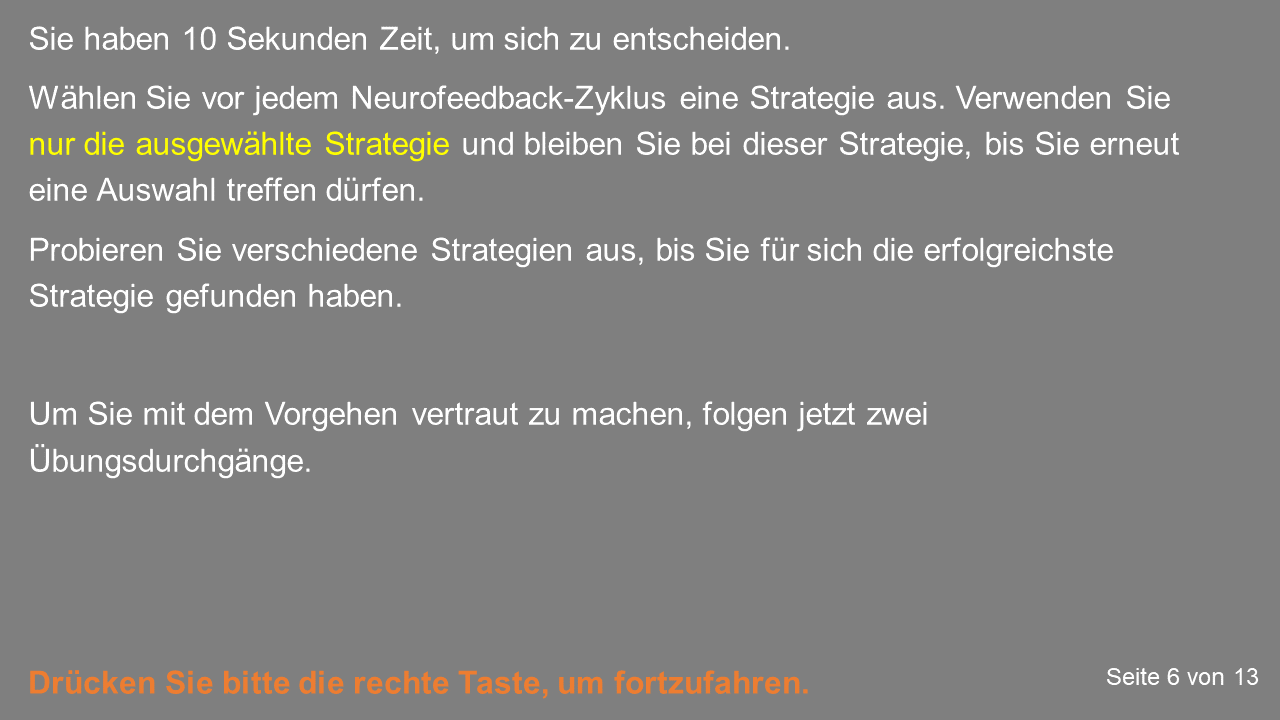


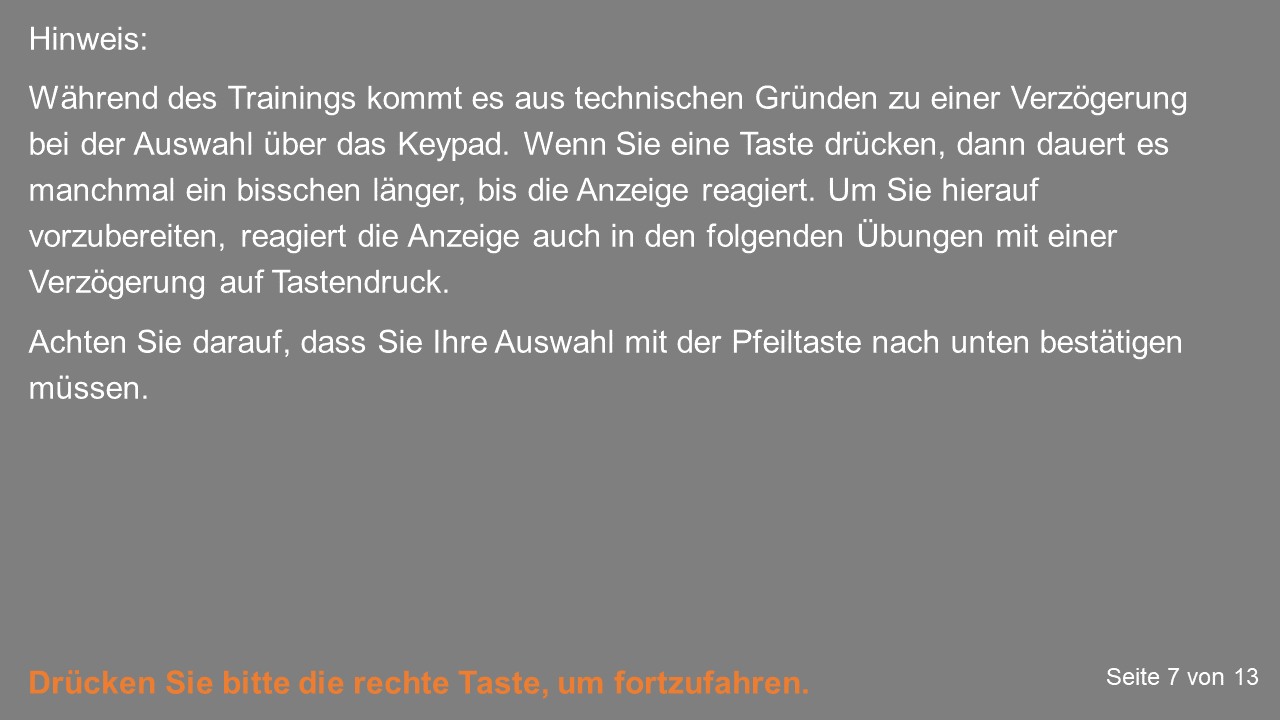


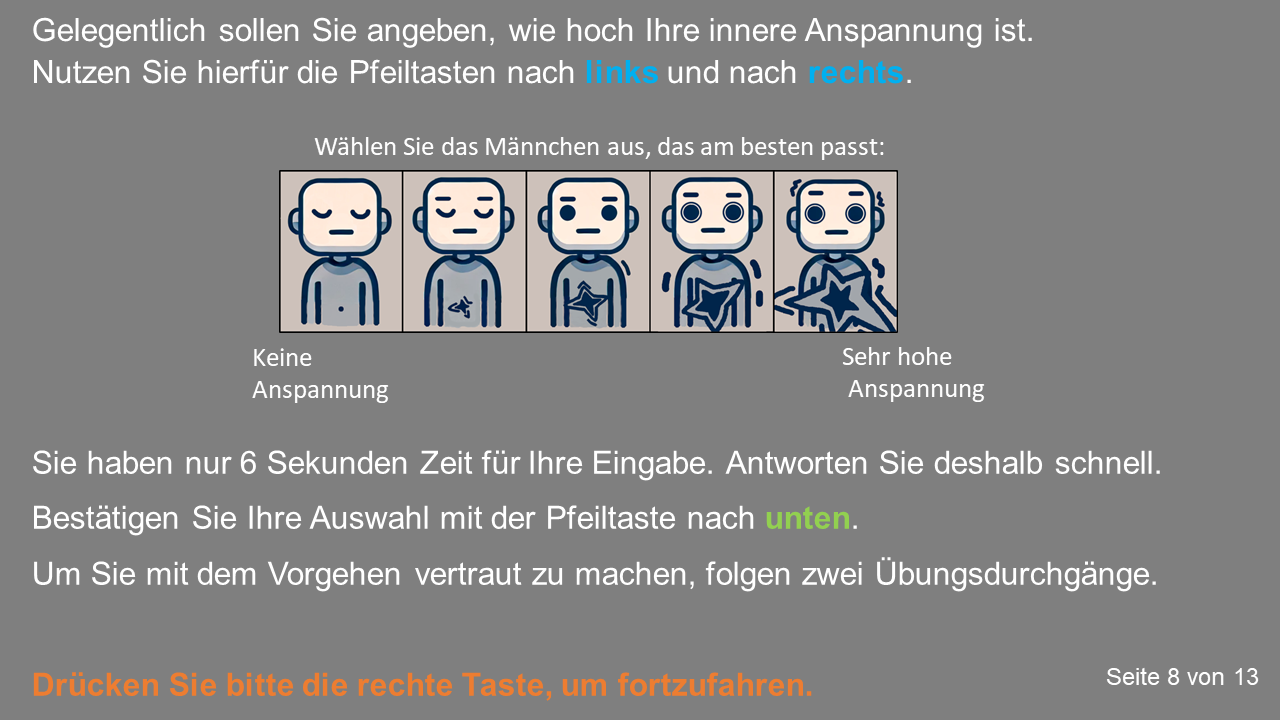


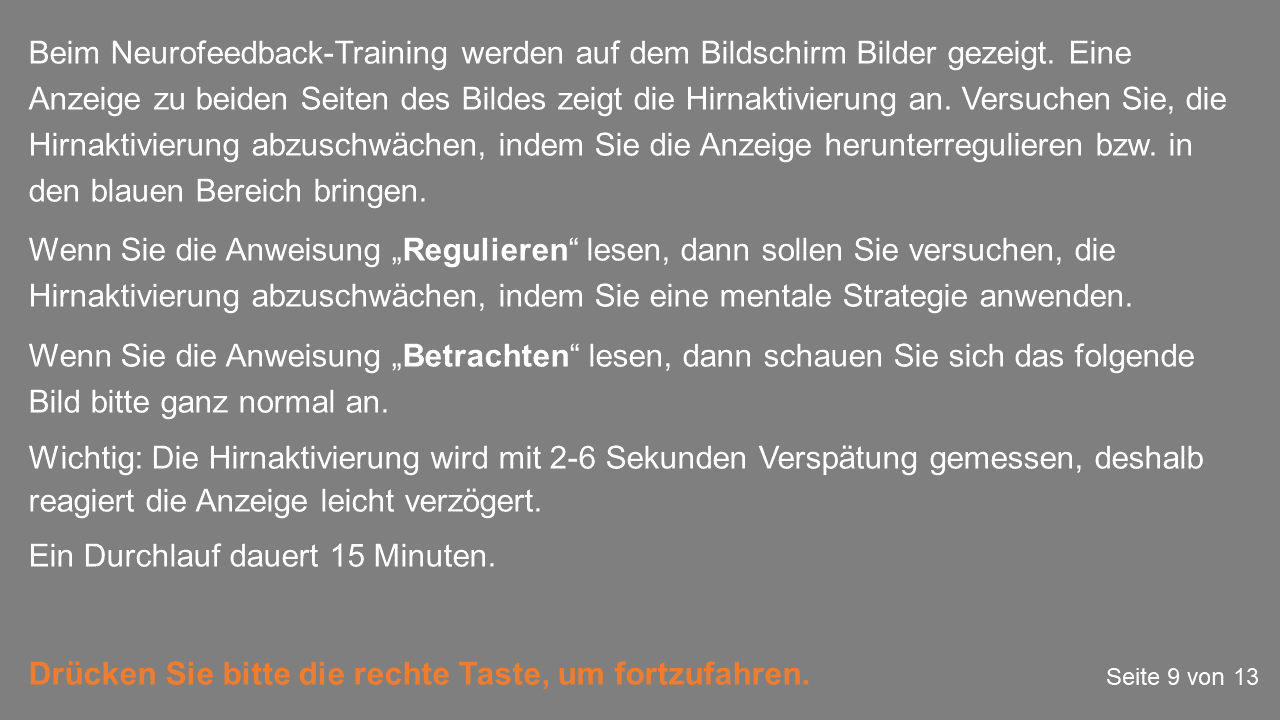


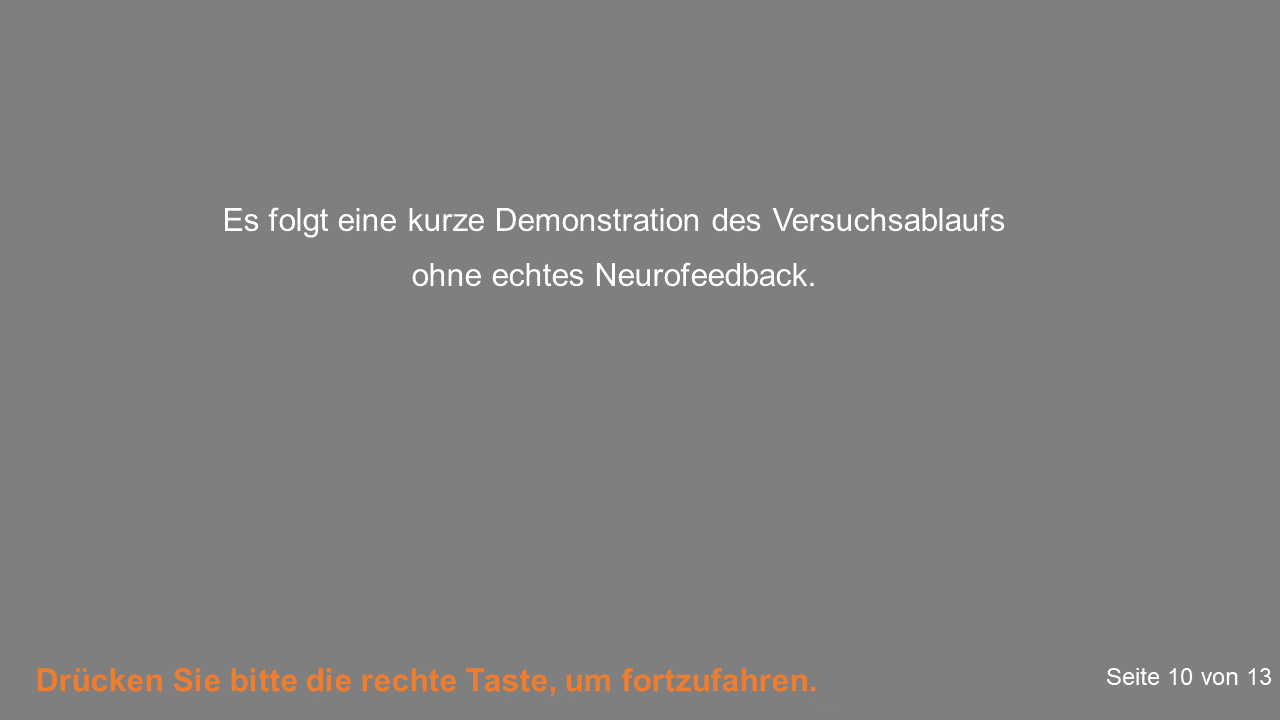


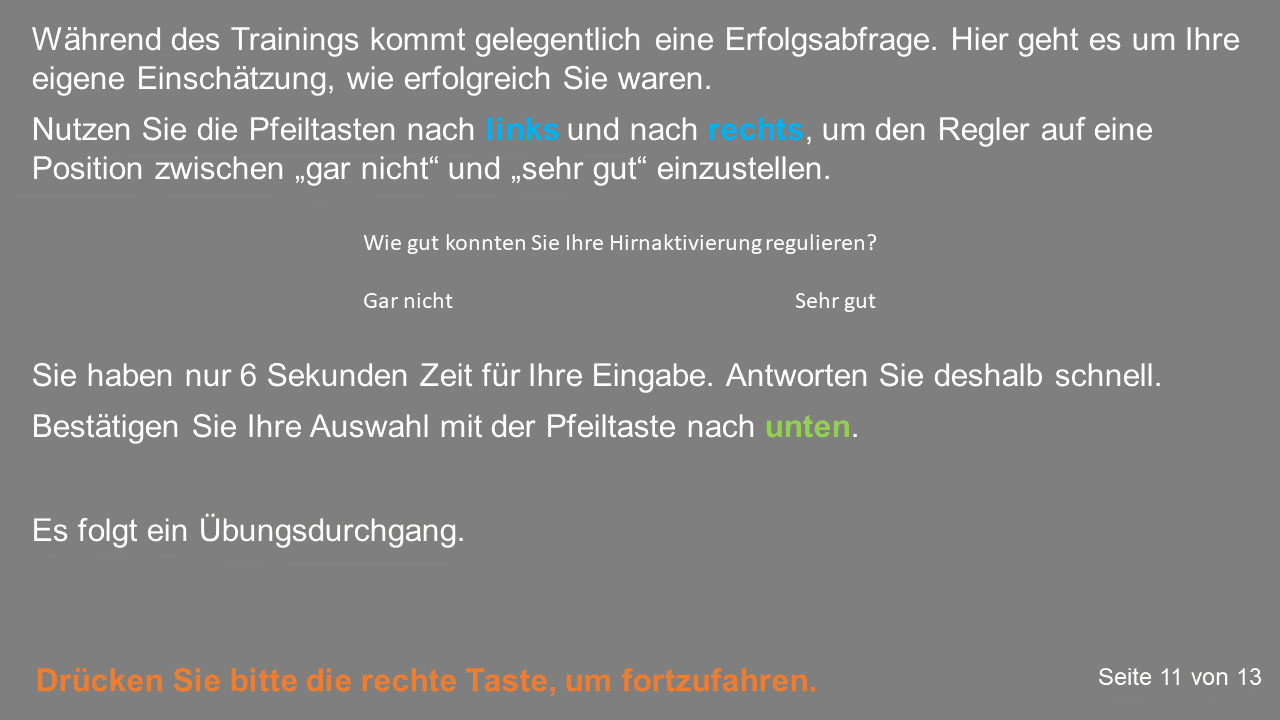


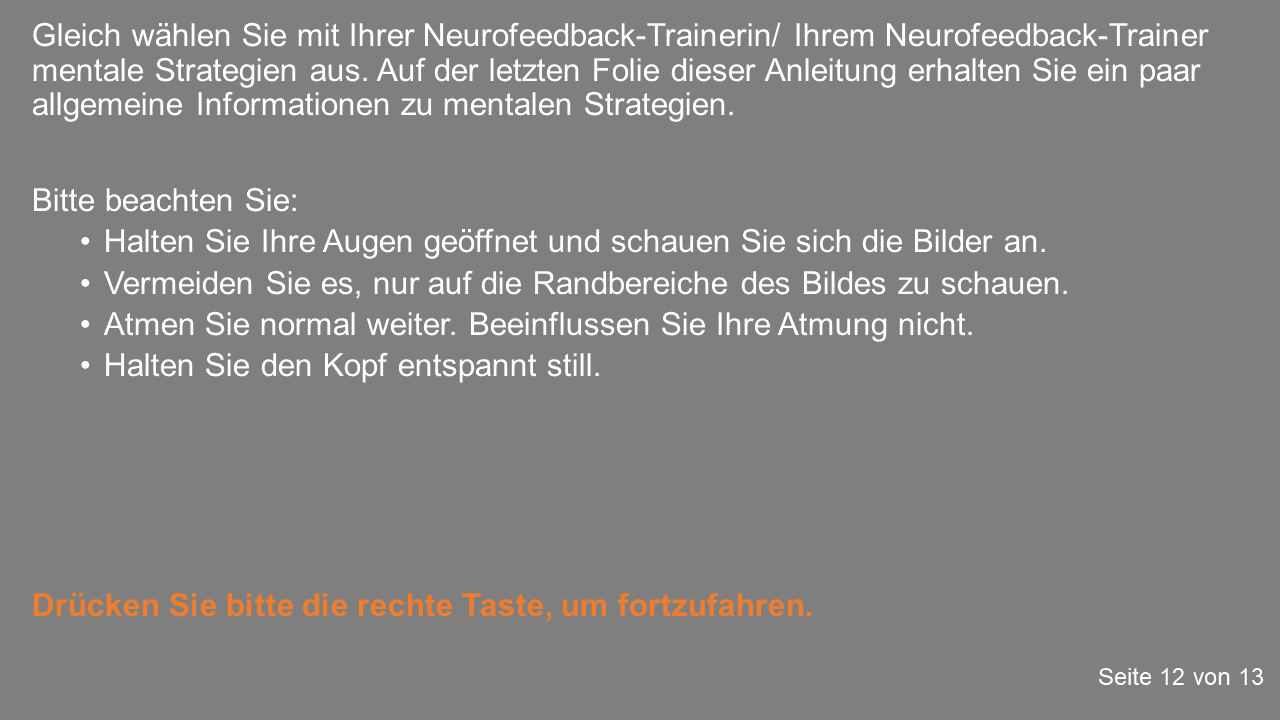


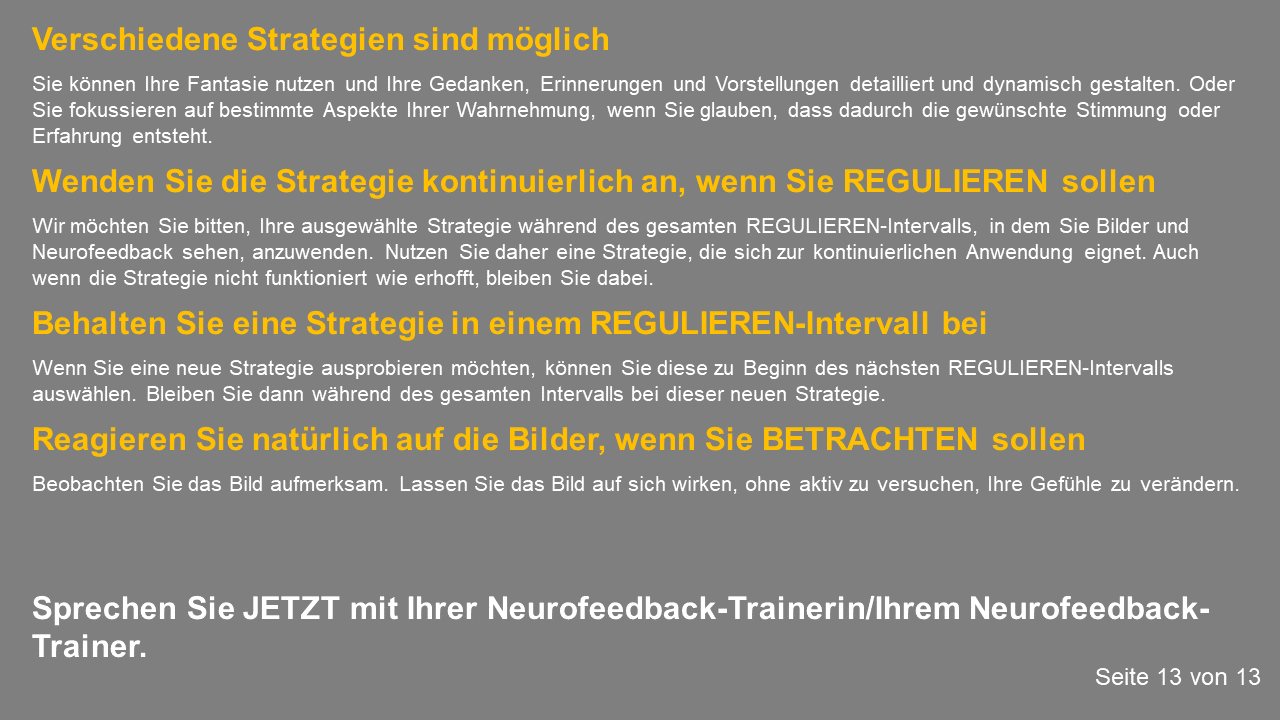

Supplement: Supplementary file 1 — Supplementary Material 1 [file 12888_2025_7000_MOESM1_ESM.docx]
